# Pol μ ribonucleotide insertion opposite 8-oxodG facilitates the ligation of premutagenic DNA repair intermediate

**DOI:** 10.1038/s41598-020-57886-y

**Published:** 2020-01-22

**Authors:** Melike Çağlayan

**Affiliations:** Department of Biochemistry and Molecular Biology, University of Florida, Gainesville, FL 32610 USA

**Keywords:** DNA, DNA repair enzymes, Biochemistry, Enzymes, Ligases

## Abstract

DNA polymerase (pol) μ primarily inserts ribonucleotides into a single-nucleotide gapped DNA intermediate, and the ligation step plays a critical role in the joining of noncomplementary DNA ends during nonhomologous end joining (NHEJ) for the repair of double-strand breaks (DSBs) caused by reactive oxygen species. Here, we report that the pol μ insertion products of ribonucleotides (rATP or rCTP), instead of deoxyribonucleotides, opposite 8-oxo-2′-deoxyguanosine (8-oxodG) are efficiently ligated and the presence of Mn^2+^ stimulates this coupled reaction *in vitro*. Moreover, our results point to a role of pol μ in mediating ligation during the mutagenic bypass of 8-oxodG, while 3′-preinserted noncanonical base pairs (3′-rA or 3′-rC) on NHEJ repair intermediates compromise the end joining by DNA ligase I or the DNA ligase IV/XRCC4 complex.

## Introduction

The concentration of ribonucleoside triphosphates (rNTPs) in the intracellular nucleotide pool is higher than that of deoxyribonucleotides (dNTPs), and the insertion of ribonucleotides into genomic DNA can hinder replication and transcription^1–4^. DNA polymerase (pol) μ, which lacks proofreading activity, inserts both dNTPs and rNTPs in a template-dependent manner during nonhomologous end joining (NHEJ) in the repair of double-strand breaks (DSBs) that are generated by ionizing radiation or reactive oxygen species as well as endogenous DNA metabolic processes^5–9^. Pol μ displays the lowest discrimination for the sugar moiety of the nucleic acid strand and shows a normal active site geometry lacking a ‘steric gate’ amino acid side chain compared to those of other X family DNA polymerases with greater discrimination, such as pol β^10–14^. Pol μ contributes to the general error-prone bypass of certain types of DNA lesions, such as 8-oxo-2′-deoxyguanosine (8-oxodG)^15–19^. Very recently, the precatalytic ternary complex structure of the pol μ active site in lesion bypass of a template 8-oxodG has been shown^20^. Almost all DNA polymerases prefer magnesium (Mg^2+^) over the other divalent metal ions for catalysis, and manganese (Mn^2+^) is considered to be a metal mutagen^21–24^. It has been shown that pol μ is activated at physiological Mn^2+^ concentrations *in vitro*, and the unusual Mn^2+^ preference of pol μ improves the efficiency of NHEJ with increased ribonucleotide insertion rate^25^. Recently, the noncanonical mechanism of ribonucleotide insertion into a single-nucleotide gapped NHEJ repair intermediate by pol μ favoring Mn^2+^ over Mg^2+^ has been reported^26^.

As the favored pathway for DSB repair, classical NHEJ involves protein-protein interactions between several components of the repair machinery^27–29^. In this process, the recognition and processing of broken DNA ends require the Ku 70/80 heterodimer in conjunction with the DNA-dependent protein kinase catalytic subunit, the DNA ligase IV/X-ray cross complementing protein 4 (XRCC4) complex, and Artemis^30^. During this process, pol μ, which fills gaps by nucleotide insertion, is known to play an important role in resolving DSBs^31,32^. The impact of the ligation step on NHEJ fidelity and the significance of DNA ligase IV in sensing diverse strand breaks after ionizing radiation have also been reported^33,34^. Our previous work revealed that pol μ Watson-Crick-like dG:dT insertion facilitates the ligation of strand break repair intermediates, suggesting the accommodation of the coupled reaction by pol μ and DNA ligase during NHEJ^35^, similar to the well-known substrate channeling mechanism in which DNA repair intermediates are transferred in a highly coordinated manner during the base excision repair (BER) pathway^36,37^. Recently, it has been proposed in a triple strand break reaction model that the ribonucleotides primarily inserted by pol μ promote ligation during the repair of chromosome breaks *in vivo*^38^.

In this study, we investigated the impact of pol μ ribonucleotide vs deoxyribonucleotide insertion into a model DSB repair substrate with a single-nucleotide gap as well as the effect of the metal ion (Mg^2+^ vs Mn^2+^) on ligation by DNA ligase I or the DNA ligase IV/XRCC4 complex. We showed that the pol μ ribonucleotide insertion products of rATP and rCTP opposite 8-oxodG or dG are efficiently ligated, and the presence of Mn^2+^ stimulates this coupled reaction *in vitro*. Moreover, the ligation of the nicked substrate with preinserted ribonucleotides (3′-rA or 3′-rC opposite 8-oxodG or dG) is compromised by subtle changes in the structure of the DNA ends. Overall, the results indicate a significant role of ribonucleotide insertion and the requirement of pol μ for efficient ligation of the NHEJ repair intermediate during the coupled double-strand break repair.

## Results

### Ligation of pol μ deoxyribonucleotide vs ribonucleotide insertion products during mutagenic bypass of template 8-oxodG

To understand the ligation of the repair intermediates by DNA ligase after pol μ-mediated deoxyribonucleotide vs ribonucleotide insertion, we first measured dNTP (dATP or dCTP) vs rNTP (rATP or rCTP) insertion coupled with ligation in a reaction mixture containing pol μ and DNA ligase I or the DNA ligase IV/XRCC4 complex (Supplementary Scheme 1). For this purpose, we used the model end joining DNA substrate with a single-nucleotide gap and a template 8-oxodG (Supplementary Table 1).

**Figure 1:**
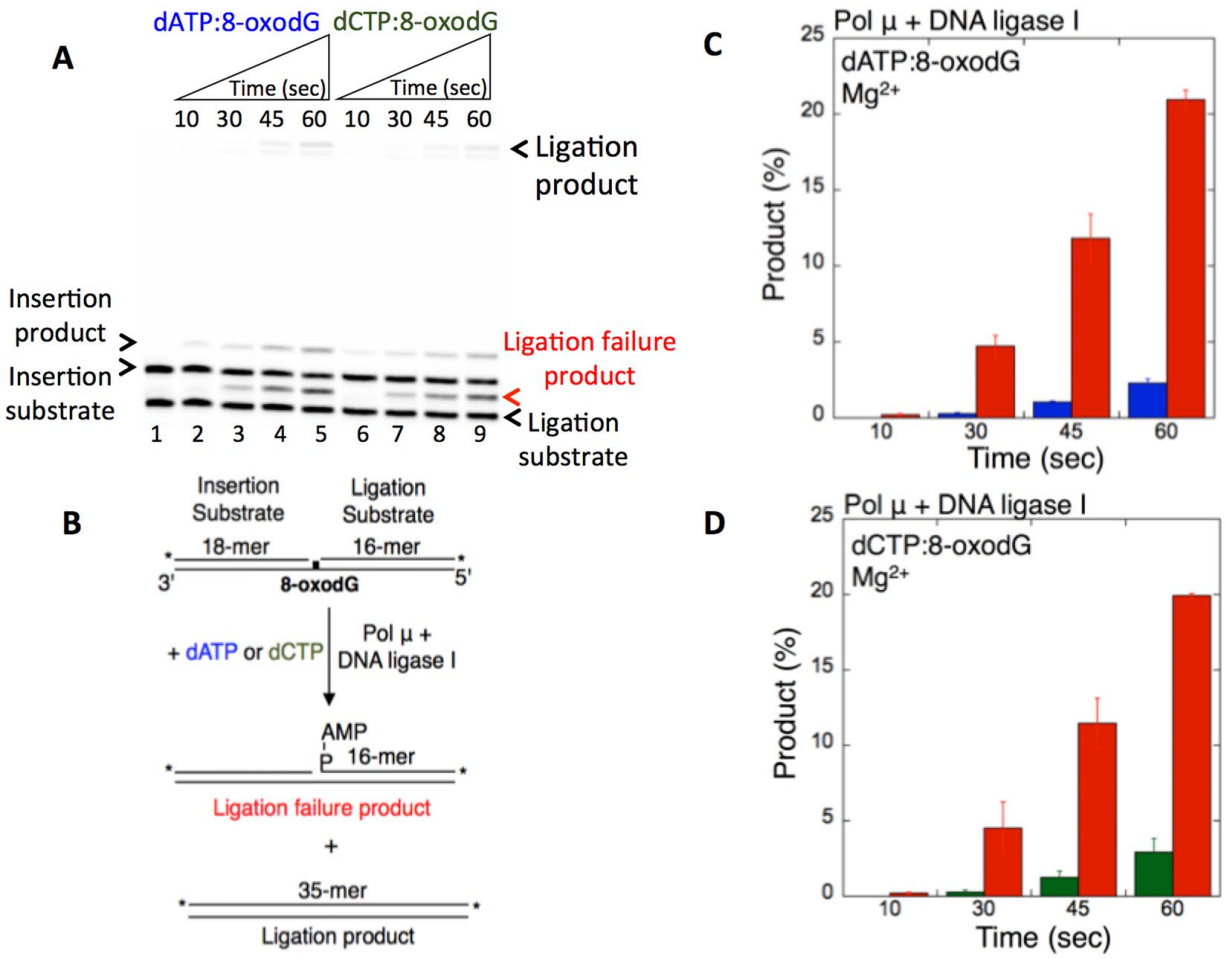
Pol µ dATP and dCTP insertion opposite 8-oxodG coupled with ligation by DNA ligase I in the presence of Mg^2+^. (**A**) Lane 1 is the minus enzyme control for the one nucleotide gapped DNA substrate with template 8-oxodG. Lanes 2–5 and 6–9 are the coupled reaction products in the presence of dATP or dCTP, respectively, and correspond to time points of 10, 30, 45, and 60 sec. The scheme illustrates the DNA substrates and reaction products observed in the coupled assay. (**B**,**C**) Graphs show time-dependent changes in the products of ligation (blue and green) and ligation failure (red). The data represent the average of three independent experiments ± SD. Corresponding uncropped gel image is shown in Supplementary Fig. 15.

**Figure 2:**
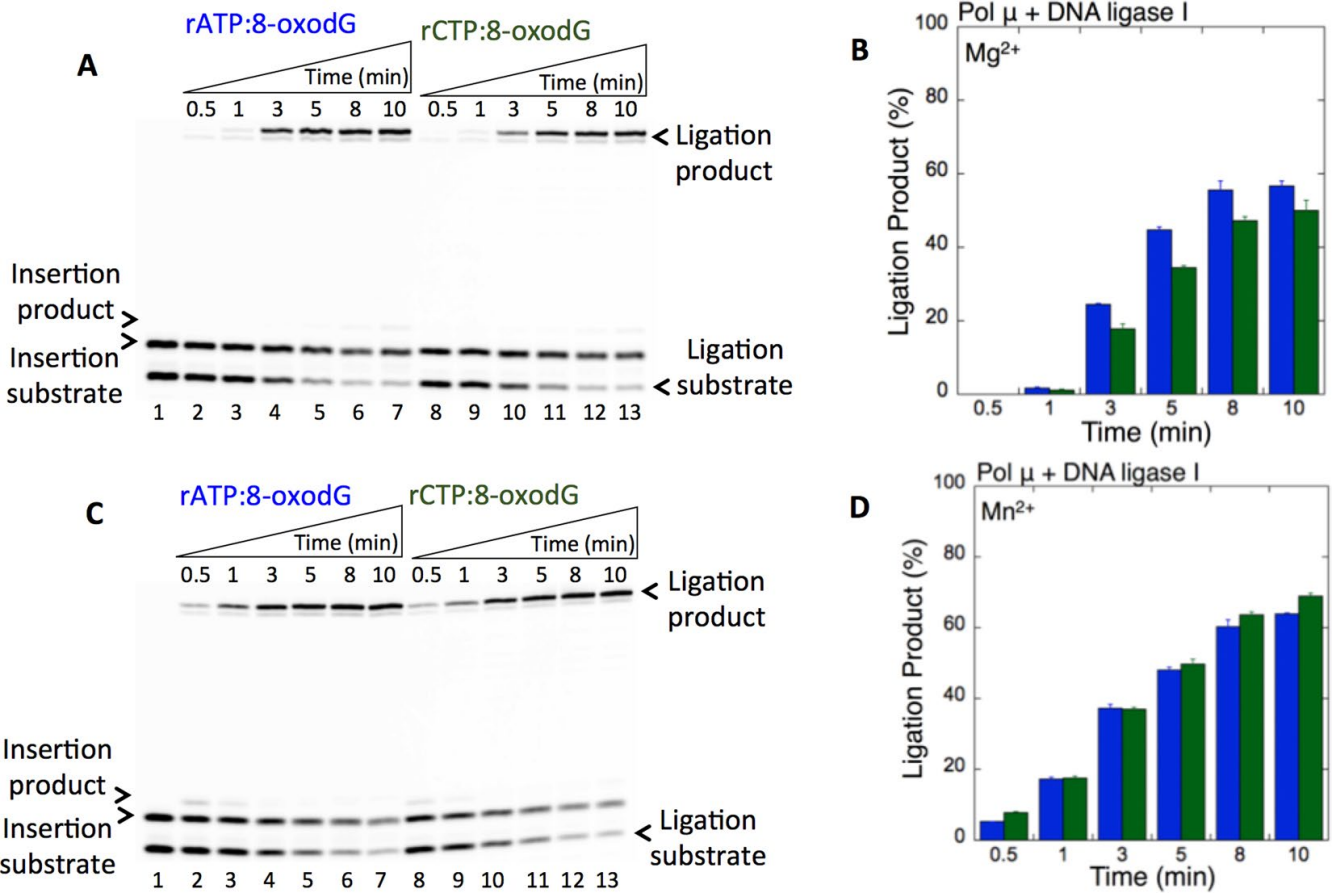
Pol µ rATP and rCTP insertion opposite 8-oxodG coupled with ligation by DNA ligase I in the presence of Mg^2+^ vs Mn^2+^. (**A**,**C**) In both panels, lane 1 is the minus enzyme control for the one nucleotide gapped DNA substrate with template 8-oxodG. Lanes 2–7 and 8–13 are the coupled reaction products in the presence of rATP or rCTP, respectively, and correspond to time points of 0.5, 1, 3, 5, 8, and 10 min. (**B**,**D**) Graphs show time-dependent changes in the products of ligation (blue for rATP:8-oxodG and green for rCTP:8-oxodG). The data represent the average of three independent experiments ± SD. Corresponding uncropped gel images are shown in Supplementary Fig. 16.

We observed that pol μ dATP or dCTP insertion opposite 8-oxodG confounds the ligation and results in ligation failure (Fig. 1A, lanes 2–5 and 6–9), as revealed by the time-dependent (10–60 sec) change in the formation of the 5′-adenylate product, *i*.*e*., AMP addition to the 5′-end of the substrate (Fig. 1C,D) obtained in a coupled BER assay (Fig. 1B). For longer time points (0.5–10 min), we obtained both ligation and a stable amount of ligation failure products after pol μ dATP and dCTP insertions opposite 8-oxodG in a coupled reaction including DNA ligase I or the DNA ligase IV/XRCC4 complex (Supplementary Fig. 1). On the other hand, the repair intermediate with a 3′-ribonucleotide inserted by pol μ bypassing 8-oxodG is efficiently ligated. In this case, pol μ rATP and rCTP insertions opposite 8-oxodG enabled ligation (Fig. 2A), and only ligation products were observed (Fig. 2B).

### Impact of Mg^2+^ vs Mn^2+^ on the ligation of pol μ ribonucleotide insertion products during mutagenic bypass of 8-oxodG

We next tested the idea that Mn^2+^, known as a mutagenic metal ion that improves the efficiency of NHEJ, might enable the more efficient ligation of the pol μ ribonucleotide insertion products^25^. For this purpose, the effect of Mg^2+^ vs Mn^2+^ on pol μ rATP or rCTP insertion coupled with ligation was evaluated.

**Figure 3:**
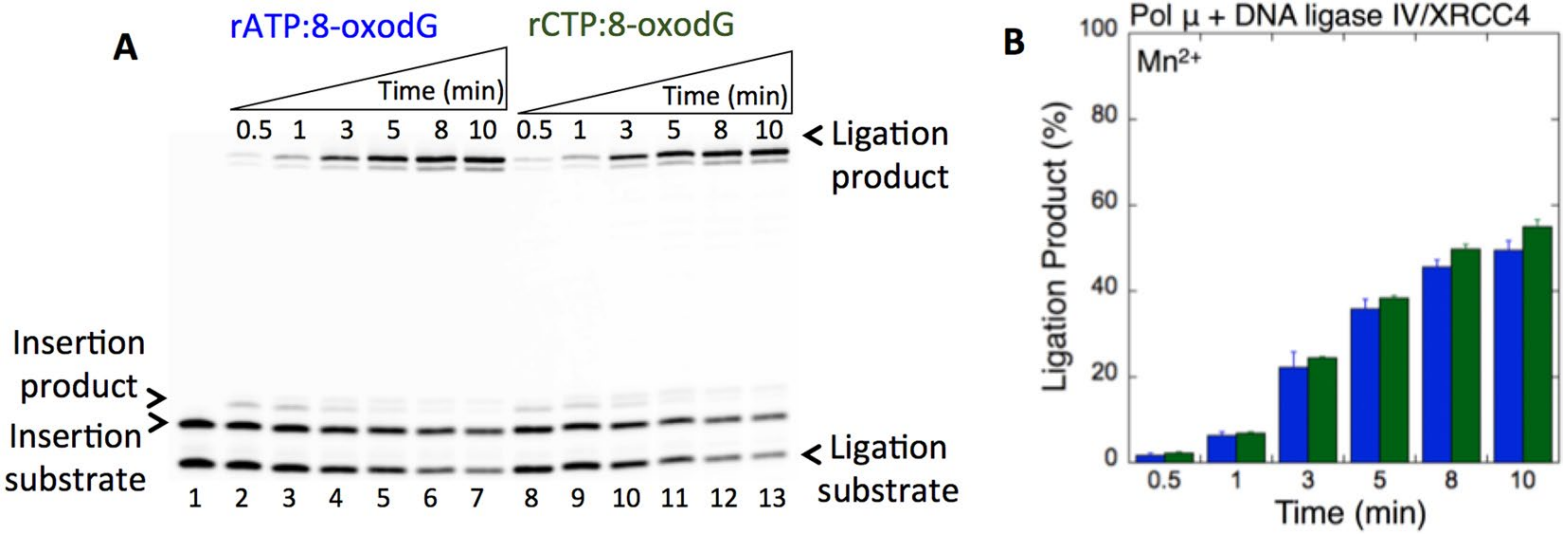
Pol µ rATP and rCTP insertion opposite 8-oxodG coupled with ligation by DNA ligase IV/XRCC4 complex in the presence of Mn^2+^. (**A**) Lane 1 is the minus enzyme control for the one nucleotide gapped DNA substrate with template 8-oxodG. Lanes 2–7 and 8–13 are the coupled reaction products in the presence of rATP or rCTP, respectively, and correspond to time points of 0.5, 1, 3, 5, 8, and 10 min. (**B**) Graph shows time-dependent changes in the products of ligation (blue for rATP:8-oxodG and green for rCTP:8-oxodG). The data represent the average of three independent experiments ± SD. Corresponding uncropped gel image is shown in Supplementary Fig. 17.

In the presence of Mg^2+^, as described above, we obtained the ligation products after pol μ rATP and rCTP insertions (Fig. 2A,B). Similarly, in the presence of Mn^2+^, the products of pol μ insertion of rATP (Fig. 2C, lanes 2–7) or rCTP (Fig. 2C, lanes 8–13) opposite 8-oxodG were efficiently ligated (Fig. 2D). However, in this case, the ligation products were slightly higher and accumulated at the earliest time point (30 sec), in contrast to the appearance of ligation products at later time points (3–10 min) in the presence of Mg^2+^ (Supplementary Fig. 2). Similarly, we obtained ligation of pol μ rATP (Fig. 3A, lanes 2–7) and rCTP (Fig. 3A, lanes 8–13) insertions opposite 8-oxodG by the DNA ligase IV/XRCC4 complex (Fig. 3B). No significant difference was observed in the amount of ligation products between DNA ligase I vs the DNA ligase IV/XRCC4 complex (Supplementary Fig. 3).

**Figure 4:**
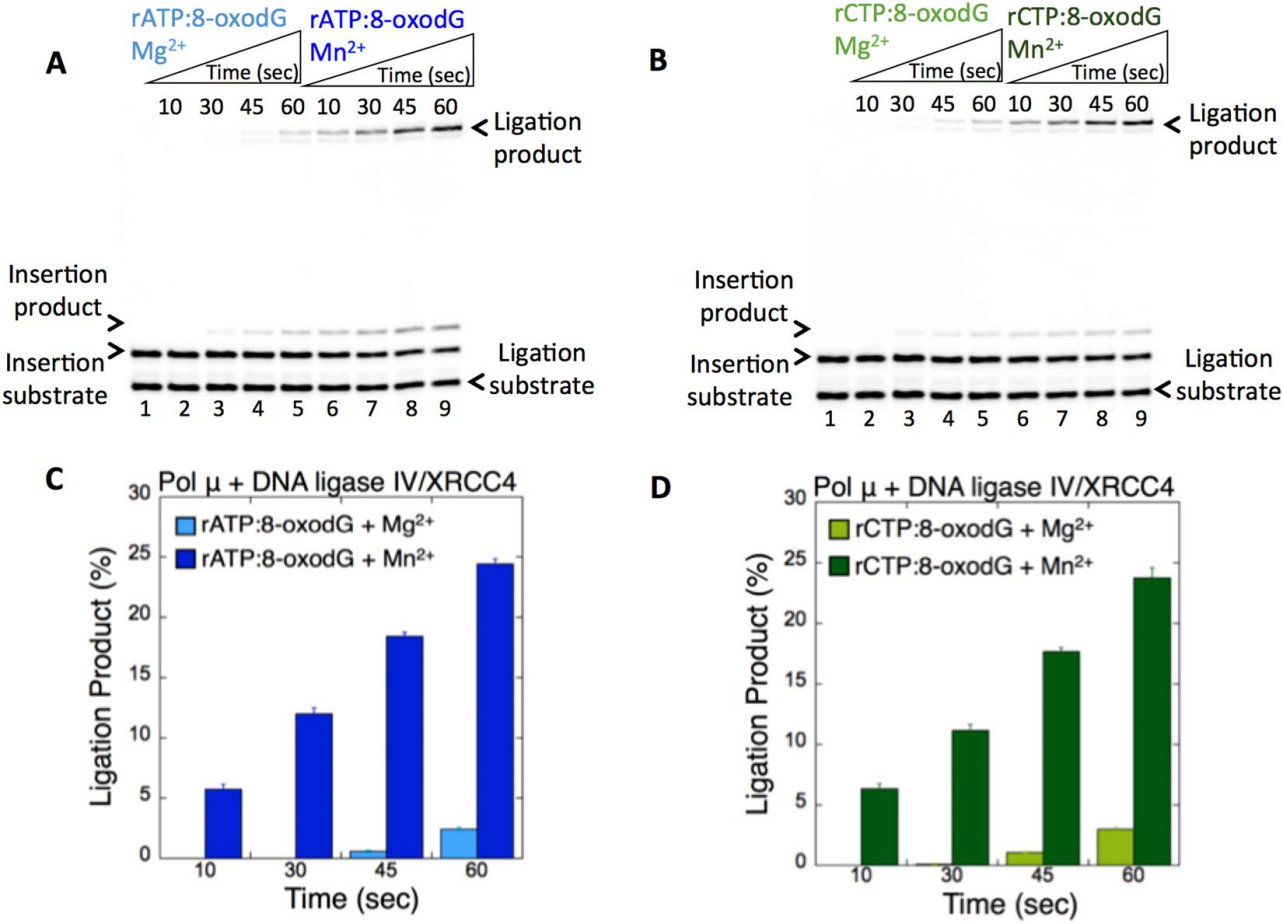
Pol µ rATP and rCTP insertion opposite 8-oxodG coupled with ligation by DNA ligase IV/XRCC4 complex in the presence of Mg^2+^ vs Mn^2+^. (**A**,**B**) In both panels, lane 1 is the minus enzyme control for the one nucleotide gapped DNA substrate with template 8-oxodG. Lanes 2–5 and 6–9 are the coupled reaction products in the presence of rATP and Mg^2+^ vs Mn^2+^ or in the presence of rCTP and Mg^2+^ vs Mn^2+^, respectively, and correspond to time points of 10, 30, 45, and 60 sec. (**C**,**D**) Graphs show the comparisons for the time-dependent changes in the products of ligation (blue for rATP:8-oxodG and green for rCTP:8-oxodG) in the presence of Mg^2+^ vs Mn^2+^. The data represent the average of three independent experiments ± SD. Corresponding uncropped gel images are shown in Supplementary Fig. 18.

To further understand the stimulatory effect of Mn^2+^ on pol μ ribonucleotide insertion coupled with ligation as described above, we repeated these coupled reactions for the early incubation time points (10–60 sec). For both pol μ rATP:8-oxodG (Fig. 4A, lanes 2–5 vs 6–9) and rCTP:8-oxodG (Fig. 4B, lanes 2–5 vs 6–9) insertions, the amounts of ligation products were ~5-fold higher in the presence of Mn^2+^ than in the presence of Mg^2+^ (Fig. 4C,D). These results suggest a stimulatory role of Mn^2+^ in the channeling of the pol μ ribonucleotide insertion products from pol μ to the next DNA ligation step.

We also examined the ribonucleotide insertions into a single-nucleotide gap with a template 8-oxodG in a reaction mixture including pol μ alone (Supplementary Scheme 2) and obtained the differences in the insertion efficiency of rATP and rCTP in the presence of Mg^2+^ vs Mn^2+^ (Supplementary Fig. 4). These results showed that the ligation products observed in the coupled reactions including both pol μ and DNA ligase (Fig. 2) are due to the insertions by pol μ favoring Mn^2+^ over Mg^2+^ and their subsequent conversion to ligation products at the same time points of the reaction incubations. In the control reactions, we also confirmed the efficient conversion of the correct pol μ dNTP insertion products, *i*.*e*., dATP opposite dT and dCTP opposite dG to complete ligation products (Supplementary Fig. 5).

### Role of NHEJ structural elements and mutagenic bypass of 8-oxodG on the ligation of pol μ ribonucleotide insertion products

**Figure 5:**
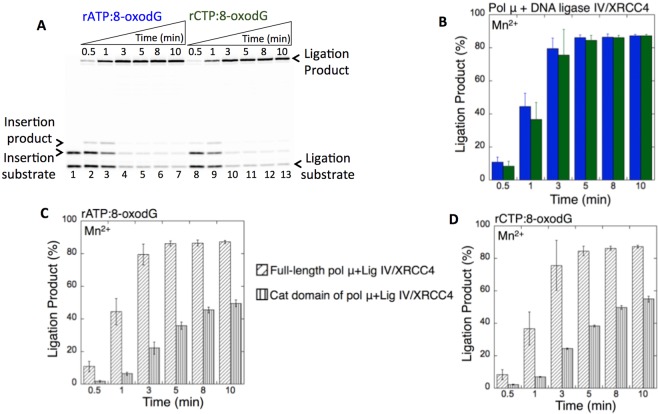
Full-length pol µ rATP and rCTP insertion opposite 8-oxodG coupled with ligation by DNA ligase IV/XRCC4 complex. (**A**) Lane 1 is the minus enzyme control for the one nucleotide gapped DNA substrate with template 8-oxodG. Lanes 2–7 and 8–13 are the coupled reaction products, and correspond to time points of 0.5, 1, 3, 5, 8, and 10 min. (**B**) Graph shows time-dependent changes in the products of ligation (blue for rATP:8-oxodG and green for rCTP:8-oxodG). The data represent the average of three independent experiments ± SD. (**C**,**D**) Graphs show the comparisons for the time-dependent changes in the products of ligation after rATP:8-oxodG and rCTP:8-oxodG insertion by the full-length vs the catalytic domain of pol µ. The data represent the average of three independent experiments ± SD. Corresponding uncropped gel image is shown in Supplementary Fig. 19.

It has been reported that the recruitment of the NHEJ complex at DNA ends during DSB repair requires the presence of pol μ and DNA ligase IV in complex with XRCC4, and the BRCT (BRCA1 C Terminus) domain of pol μ stabilizes the complex formation and promotes ligation independent of its gap-filling activity^32,39,40^. To further investigate the channeling of the ribonucleotide insertion products from pol μ to DNA ligase, we used the full-length pol μ and performed the coupled reactions in the presence of the DNA ligase IV/XRCC4 complex and Mn^2+^. Similarly, the results showed ligation of pol μ rATP (Fig. 5A, lanes 2–7) and rCTP (Fig. 5A, lanes 8–13) insertions and a time-dependent increase in the amounts of the products (Fig. 5B). However, the comparison of the coupled reactions revealed an enhanced ligation in the presence of the full-length pol μ including BRCT domain for both insertions (Fig. 5C,D). These findings suggest a coordinated hand off or interplay between pol μ and DNA ligase IV/XRCC4 complex in the multiprotein NHEJ complex for efficient ligation in the presence of ribonucleotides incorporated into DNA (Supplementary Model 1).

**Figure 6:**
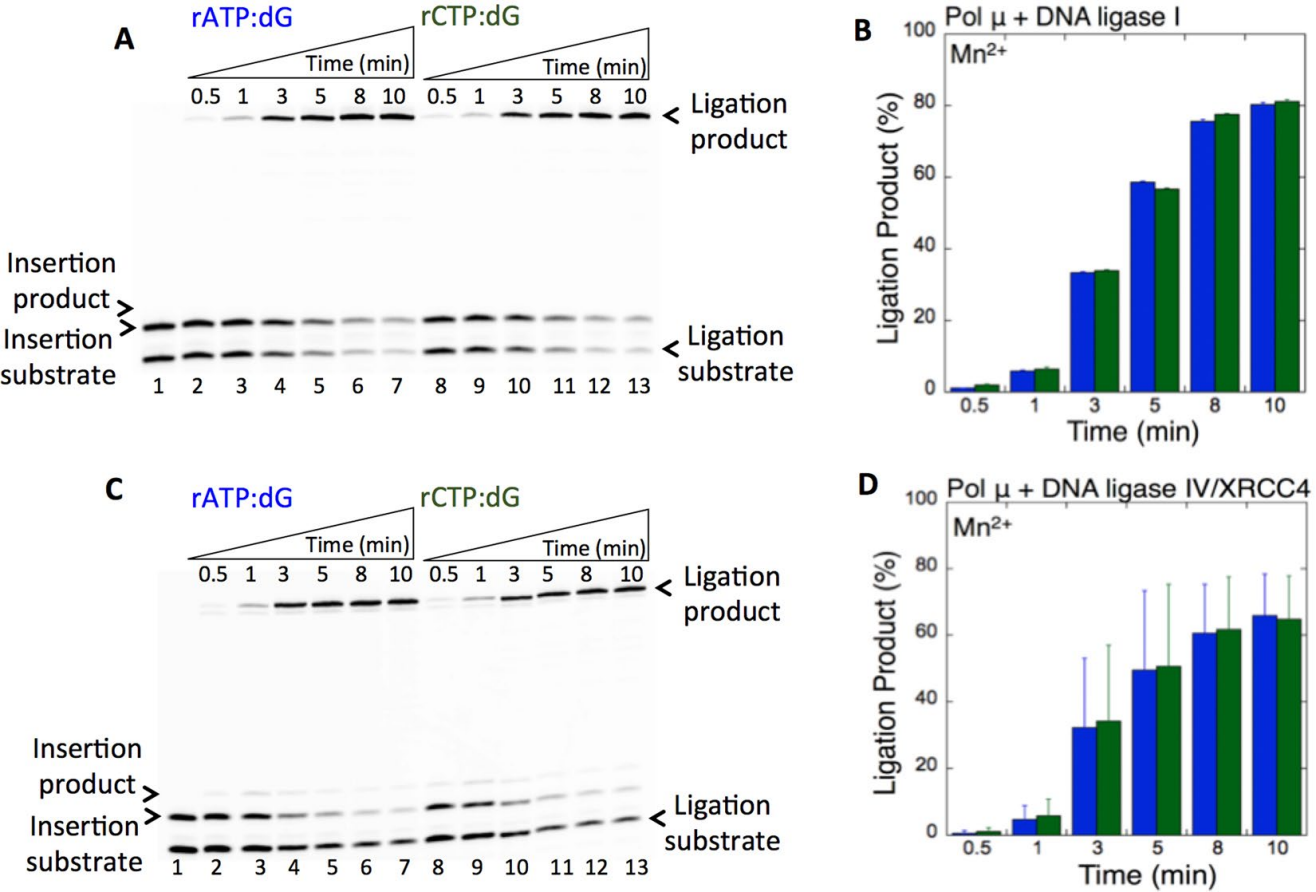
Pol µ rATP and rCTP insertion opposite dG coupled with ligation by DNA ligase I vs DNA ligase IV/XRCC4 complex. (**A**,**C**) In both panels, lane 1 is the minus enzyme control for the one nucleotide gapped DNA substrate with template dG. Lanes 2–7 and 8–13 are the coupled reaction products in the presence of rATP or rCTP, respectively, and correspond to time points of 0.5, 1, 3, 5, 8, and 10 min. (**B**,**D**) Graphs show time-dependent changes in the products of ligation (blue for rATP:dG and green for rCTP:dG). The data represent the average of three independent experiments ± SD. Corresponding uncropped gel images are shown in Supplementary Fig. 20.

Moreover, to understand the impact of pol μ mutagenic bypass of 8-oxodG on ligation, we also used a single-nucleotide gapped DNA substrate with an undamaged guanine base (dG) at a template position (Supplementary Table 1) and compared the ligation efficiency of the pol μ ribonucleotide insertion products opposite 8-oxodG vs dG in the presence of Mn^2+^. The results revealed that the products of pol μ rATP:dG (Fig. 6A,C, lanes 2–7) and rCTP:dG (Fig. 6A,C, lanes 8–13) are efficiently ligated by DNA ligase I (Fig. 6B) and the DNA ligase IV/XRCC4 complex (Fig. 6D). Similarly, no significant difference was observed in the amount of ligation products between the DNA ligases (Supplementary Fig. 6). Interestingly, the comparison of the ligation products after pol μ rATP and rCTP insertion opposite 8-oxodG vs dG also shows no significant difference between the template bases (Supplementary Figs. 7 and 8). These results suggest that the pol μ active site can accommodate both 8-oxodG and dG in a similar way that enables the channeling of the ribonucleotide mismatch-containing repair intermediate from pol μ to DNA ligase for its efficient ligation. We also confirmed differences in the pol μ insertion efficiency of rATP and rCTP into a single-nucleotide gap with a template dG in the reaction mixture including pol μ alone (Supplementary Fig. 9).

### Comparison of pol β-mediated ribonucleotide insertion coupled with ligation

It has been shown that pol β can perform inefficient ribonucleotide insertion (~4 orders of magnitude less than dNTPs) during BER, and unlike pol µ, the enzyme undergoes large conformational changes in protein subdomains upon dNTP binding or catalysis^41–43^. Moreover, the role of pol β in NHEJ has been reported^44^.

**Figure 7:**
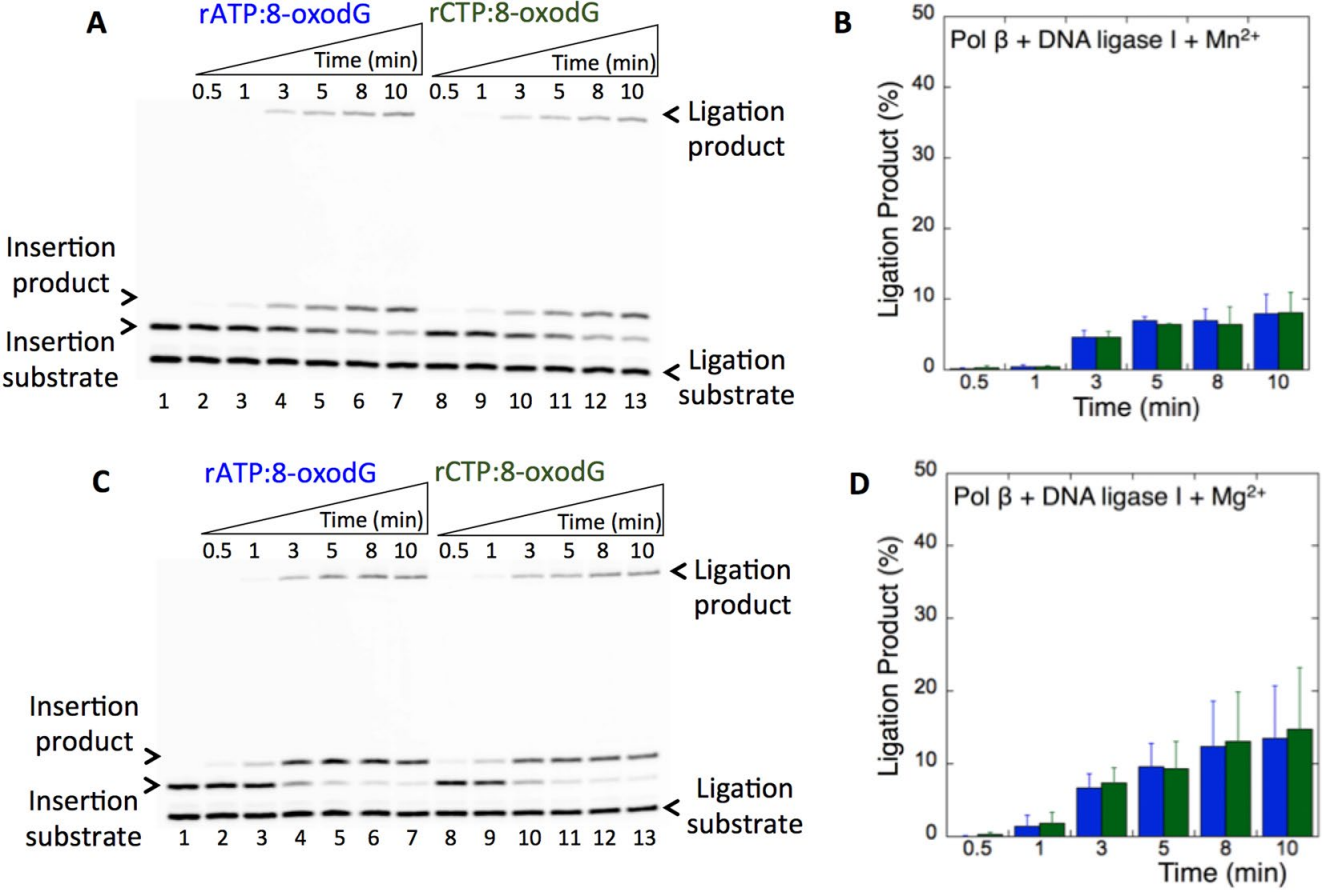
Pol β rATP and rCTP insertion opposite 8-oxodG coupled with ligation by DNA ligase I in the presence of Mg^2+^ vs Mn^2+^. (**A**,**C**) In both panels, lane 1 is the minus enzyme control for the one nucleotide gapped DNA substrate with template 8-oxodG. Lanes 2–7 and 8–13 are the coupled reaction products in the presence of rATP or rCTP, respectively, and correspond to time points of 0.5, 1, 3, 5, 8, and 10 min. (**B**,**D**) Graphs show time-dependent changes in the products of ligation (blue for rATP:dG and green for rCTP:dG). The data represent the average of three independent experiments ± SD. Corresponding uncropped gel images are shown in Supplementary Fig. 21.

In this study, we also tested the effect of pol β ribonucleotide insertion opposite 8-oxodG on ligation as described above for pol µ. We obtained the pol β insertion products of rATP:8-oxodG (Fig. 7A,C, lanes 3–7) and rCTP:8-oxodG (Fig. 7A,C, lanes 9–13) in the presence of Mg^2+^ or Mn^2+^ that were not completely ligated by DNA ligase I (Fig. 7B,D). There were ~5- and ~7-fold differences in the amount of ligation products between the coupled reactions including pol β vs pol μ in the presence of Mg^2+^ and Mn^2+^, (Supplementary Figs. 10 and 11), respectively. This inefficient channeling of the ribonucleotide-containing repair intermediate from pol β to DNA ligase could be due to differences in the mechanism of sugar discrimination between these DNA polymerases during the bypass of 8-oxodG.

### Effect of 3′-preinserted ribonucleotides on the efficiency of DNA ligation

We are also interested in examining the ligation of the repair intermediates with 3′-preinserted ribonucleotides in the presence of Mn^2+^ by DNA ligase alone (Supplementary Scheme 3). The nicked DNA substrates used here mimic the products of DNA polymerase-mediated ribonucleotide insertions and include 3′-rA or 3′-rC opposite 8-oxodG or dG (Supplementary Table 1).

**Figure 8:**
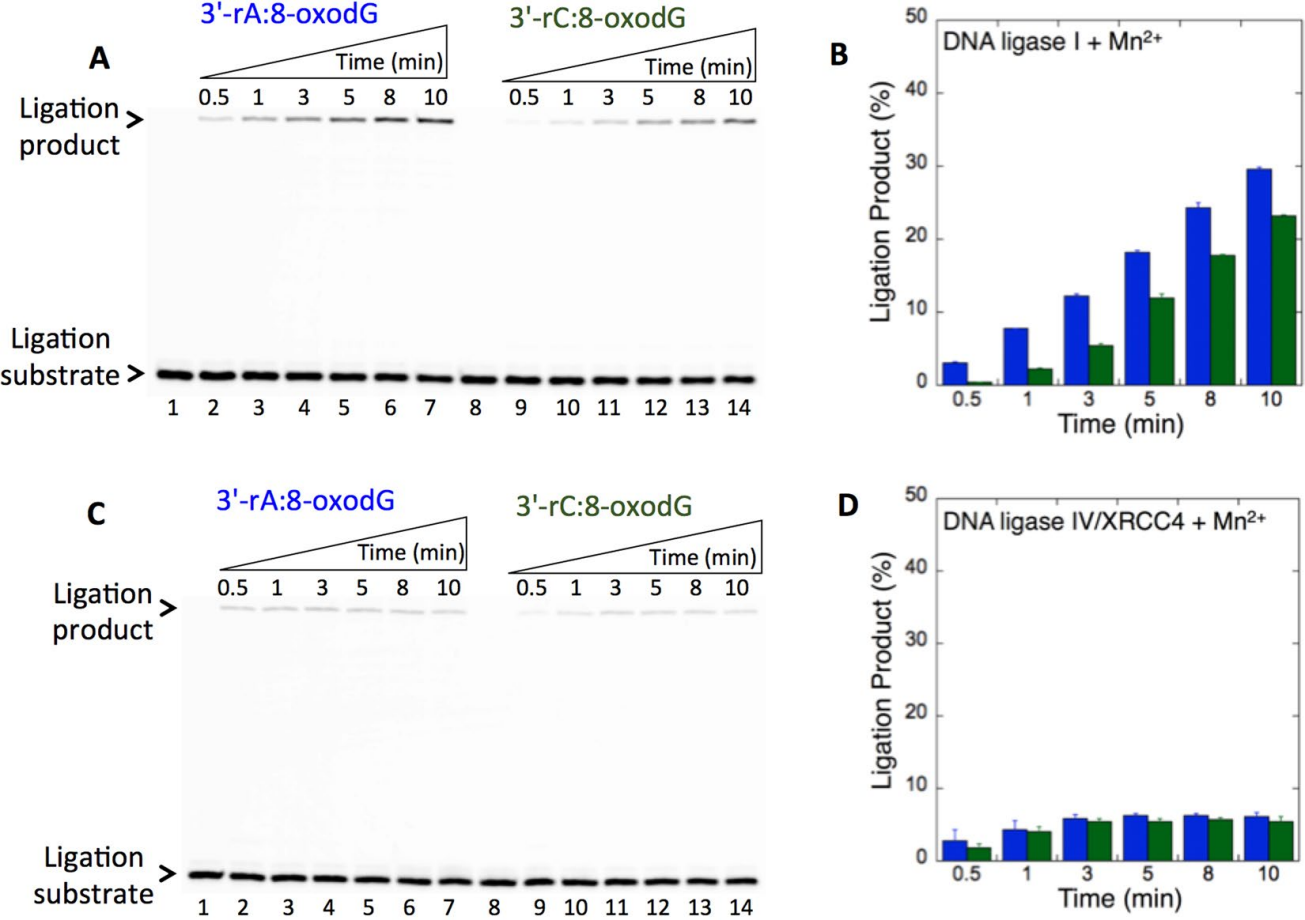
The ligation of preinserted 3′-rA and 3′-rC opposite 8-oxodG by DNA ligase I vs DNA ligase IV/XRCC4 complex. (**A**,**C**) In both panels, lanes 1 and 8 are the minus enzyme controls for the nicked DNA substrates with 3′-rA:8-oxodG and 3′-rC:8-oxodG, respectively. Lanes 2–7 and 9–14 are the ligation products in the presence of Mn^2+^, and correspond to time points of 0.5, 1, 3, 5, 8, and 10 min. (**B**,**D**) Graphs show time-dependent changes in the products of ligation (blue for 3′-rA:8-oxodG and green for 3′-rC:8-oxodG) by DNA ligase I (**B**) and DNA ligase IV/XRCC4 complex. (**D**) The data represent the average of three independent experiments ± SD. Corresponding uncropped gel images are shown in Supplementary Fig. 22.

We first tested the ligation efficiency of the nicked repair intermediates with 3′-rA or 3′-rC opposite 8-oxodG by DNA ligase I and the DNA ligase IV/XRCC4 complex. DNA ligase I was able to join the 3′-rA and 3′-rC ends (Fig. 8A, lanes 2–7 and 9–14, respectively) opposite 8-oxodG, as indicated by the time-dependent changes in the amounts of the ligation products (Fig. 8B). On the other hand, the DNA ligase IV/XRCC1 complex did not show similar activity for the nicked substrates with 3′-rA:8-oxodG (Fig. 8C, lanes 2–7) and 3′-rC:8-oxodG (Fig. 8C, lanes 9–14), and the amount of ligation products was lower (Fig. 8D). The comparison of the ligation products indicated an ~4- to 6-fold difference between the DNA ligases tested in this study (Supplementary Fig. 12).

**Figure 9:**
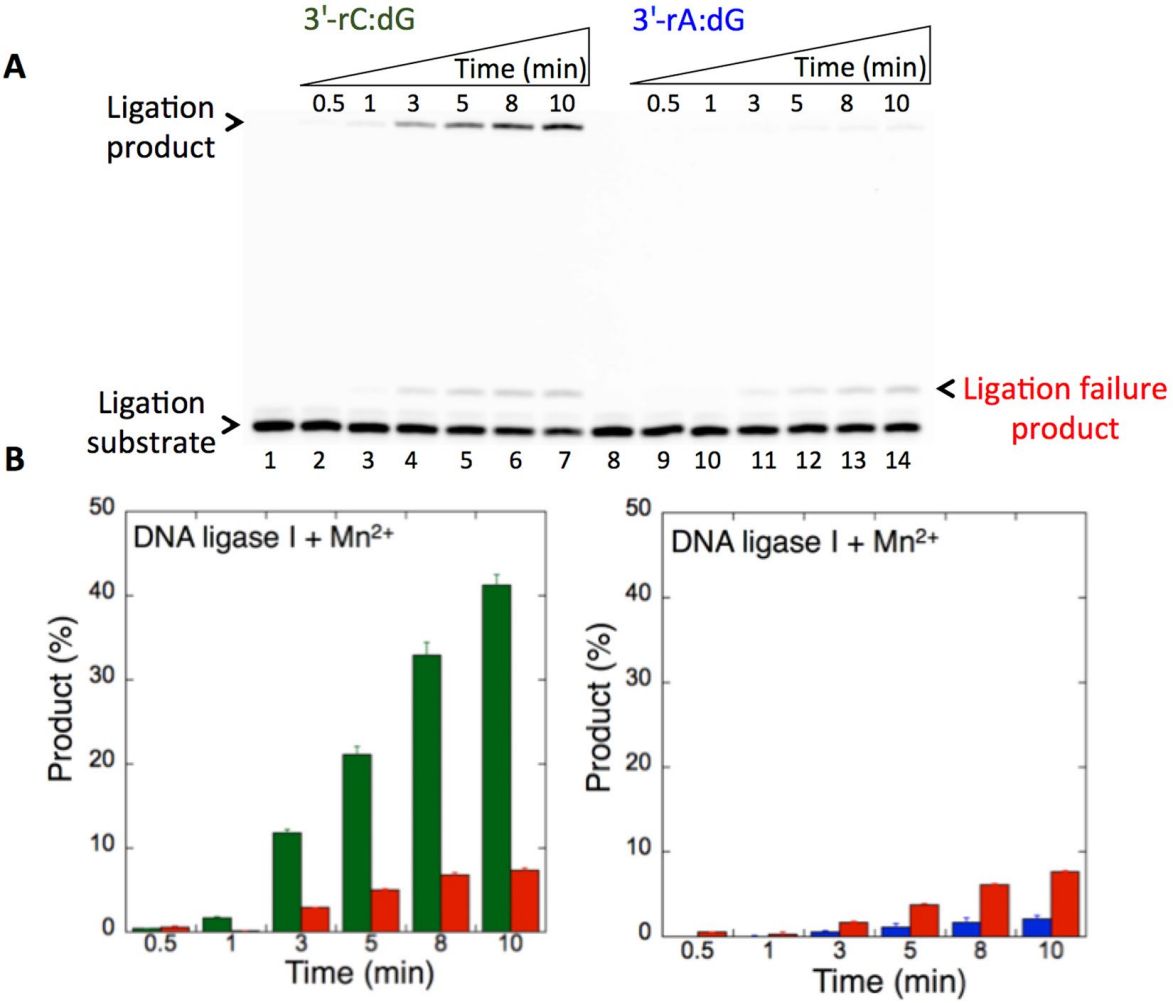
The ligation of preinserted 3′-rC and 3′-rA opposite dG by DNA ligase I. (**A**) Lanes 1 and 8 are the minus enzyme controls for the nicked DNA substrates with 3′-rC:dG and 3′-rA:dG, respectively. Lanes 2–7 and 9–14 are the ligation products in the presence of Mn^2+^, and correspond to time points of 0.5, 1, 3, 5, 8, and 10 min. (**B**) Graphs show time-dependent changes in the products of ligation (blue for 3′-rA:dG and green for 3′-rC:dG) and ligation failure (red). The data represent the average of three independent experiments ± SD. Corresponding uncropped gel image is shown in Supplementary Fig. 23.

**Figure 10:**
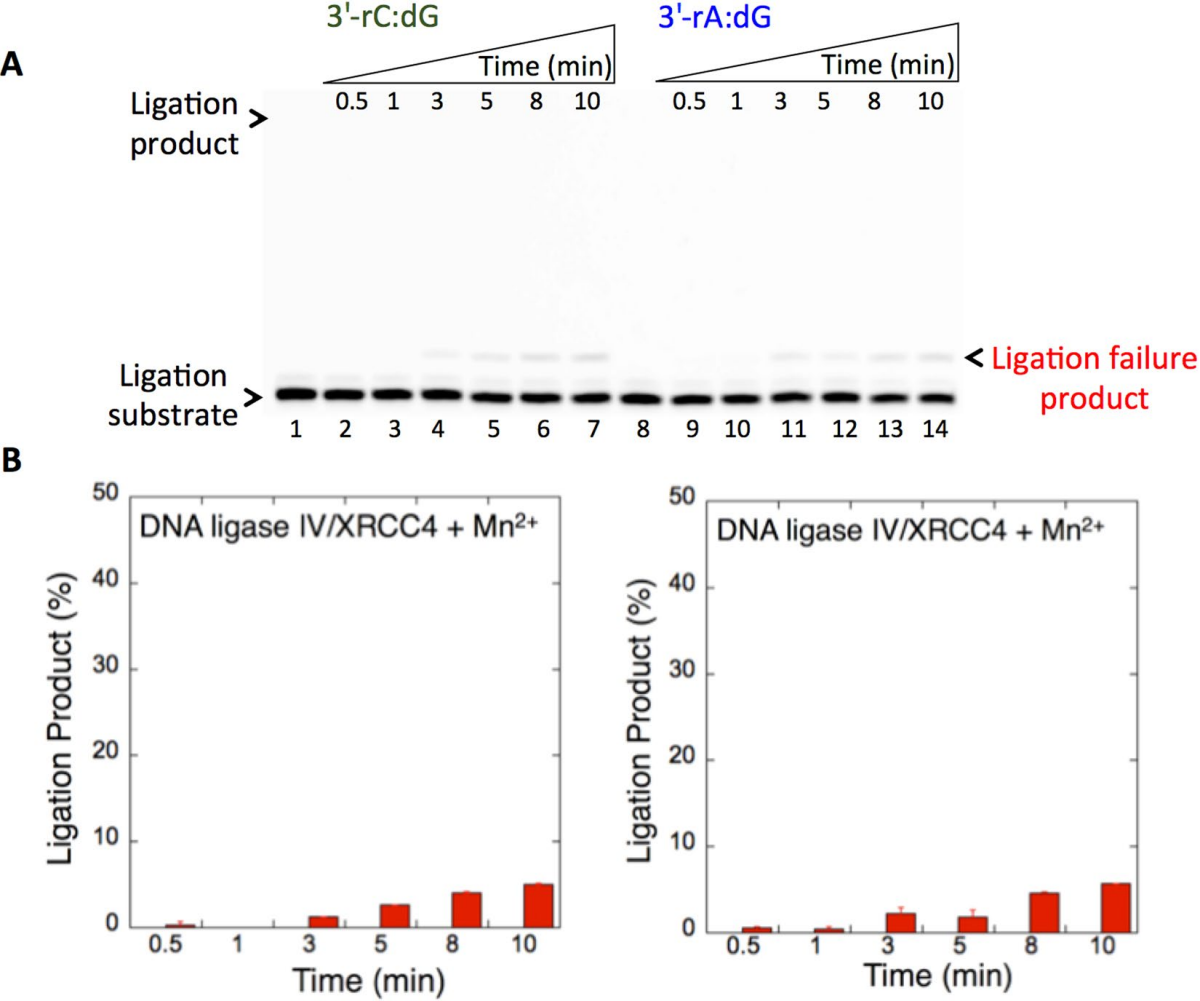
The ligation of preinserted 3′-rC and 3′-rA opposite dG by DNA ligase IV/XRCC4 complex. (**A**) Lanes 1 and 8 are the minus enzyme controls for the nicked DNA substrates with 3′-rC:dG and 3′-rA:dG, respectively. Lanes 2–7 and 9–14 are the ligation products in the presence of Mn^2+^ and correspond to time points of 0.5, 1, 3, 5, 8, and 10 min. (**B**) Graphs show time-dependent changes in the products of ligation (blue for 3′-rA:dG and green for 3′-rC:dG) and ligation failure (red). The data represent the average of three independent experiments ± SD. Corresponding uncropped gel image is shown in Supplementary Fig. 24.

We then examined the specificity of the DNA ligases for the ligation of repair intermediates with preinserted 3′-rA or 3′-rC opposite dG. Interestingly, a significant difference (~40-fold more ligation products) in the mismatch specificity of DNA ligase I was observed for the repair intermediates with 3′-rC:dG (Fig. 9A, lanes 2–7) vs 3′-rA:dG (Fig. 9A, lanes 9–14). In both mismatches, products of ligation and ligation failure appeared simultaneously (Fig. 9B). On the other hand, the DNA ligase IV/XRCC4 complex showed almost no activity against the nicked substrates with 3′-rC:dG (Fig. 10A, lanes 2–7) and 3′-rA:dG (Fig. 10A, lanes 9–14), although a negligible amount of ligation failure products accumulated at later incubation time points (Fig. 10B). The comparison of the ligation products indicated a significant difference in the end joining ability of DNA ligase I vs the DNA ligase IV/XRCC4 complex for the nicked substrates with 3′-rC:dG or 3′-rA:dG (Supplementary Fig. 13). The results suggest that the DNA ligases tested in this study could be compromised by subtle changes in the noncanonical base pairs at the 3′-end or template base of the nicked repair intermediate. Our results indicated that the presence of 8-oxodG (Fig. 8), instead of dG (Figs. 9 and 10), at the template position resulted in complete ligation with no failure. This could be due to the differences in the substrate and mismatch specificity of the DNA ligases and their end-joining ability of the non-canonical base pairs that mimic the nicked products of mismatch insertions by an error-prone DNA polymerase.

In the control reaction with the nicked DNA substrate containing 3′-preinserted correct base pairs, *i*.*e*., 3′-dA opposite dT and 3′-dC opposite dG, we confirmed the ligation over incubation time of the reaction by DNA ligase I or the DNA ligase IV/XRCC4 complex (Supplementary Fig. 14).

### The presence of pol µ mediates the ligation of ribonucleotide-containing NHEJ repair intermediates

**Figure 11:**
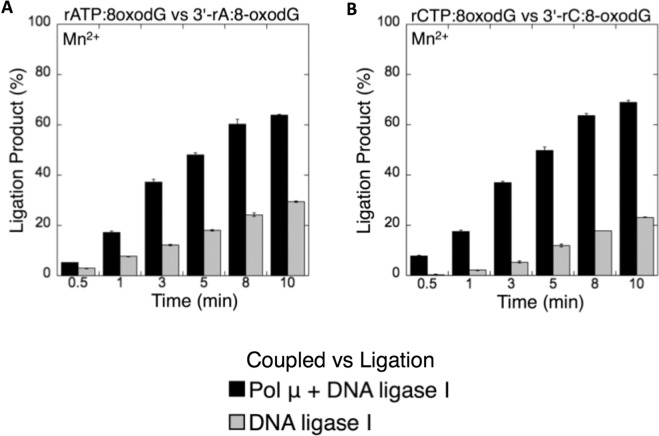
Coupled vs ligation for pol µ and DNA ligase I in the presence of template 8-oxodG. Graphs show the comparisons for the time-dependent changes in the products of ligation between the coupled and ligation reactions for rATP:8-oxodG vs 3′-rA:8-oxodG (**A**) and rCTP:8-oxodG vs 3′-rC:8-oxodG (**B**). The data represent the average of three independent experiments ± SD.

**Figure 12:**
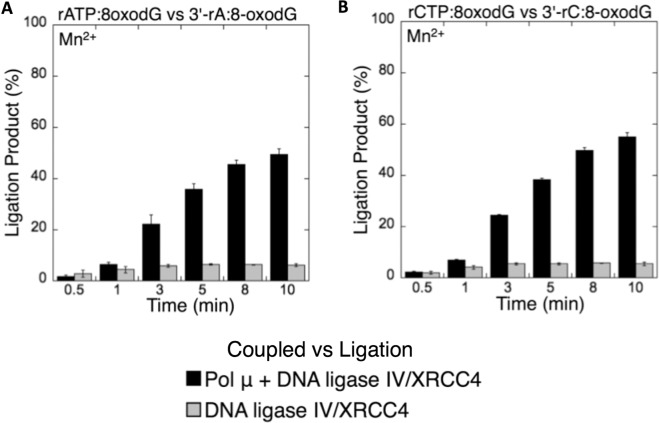
Coupled vs ligation for pol µ and DNA ligase IV/XRCC4 complex in the presence of template 8-oxodG. Graphs show the comparisons for the time-dependent changes in the products of ligation between the coupled and ligation reactions for rATP:8-oxodG vs 3′-rA:8-oxodG (**A**) and rCTP:8-oxodG vs 3′-rC:8-oxodG (**B**). The data represent the average of three independent experiments ± SD.

**Figure 13:**
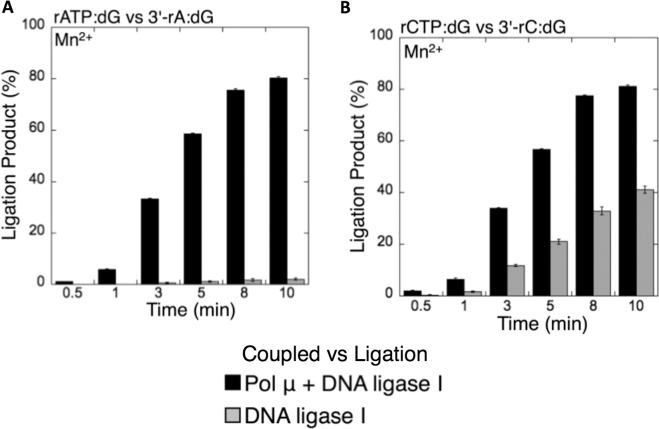
Coupled vs ligation for pol µ and DNA ligase I in the presence of template dG. Graphs show the comparisons for the time-dependent changes in the products of ligation between the coupled and ligation reactions for rATP:dG vs 3′-rA:dG (**A**) and rCTP:dG vs 3′-rC:dG (**B**). The data represent the average of three independent experiments ± SD.

**Figure 14:**
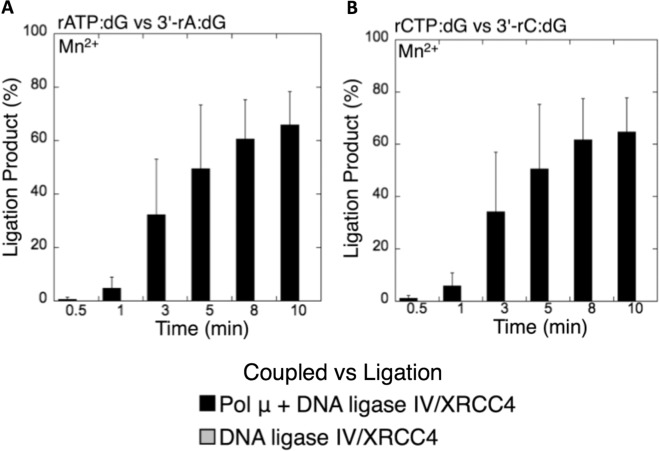
Coupled vs ligation for pol µ and DNA ligase IV/XRCC4 complex in the presence of template dG. Graphs show the comparisons for the time-dependent changes in the products of ligation between the coupled and ligation reactions for rATP:dG vs 3′-rA:dG (**A**) and rCTP:dG vs 3′-rC:dG (**B**). The data represent the average of three independent experiments ± SD.

Based on the observations described above, we finally compared the efficiency of ligation between the coupled reactions including pol µ and DNA ligase and the ligation assays containing DNA ligase alone. Overall, the results revealed that the amount of ligation products in the coupled reactions (*i*.*e*., ligation after pol µ rATP or rCTP insertion into a single-nucleotide gapped DNA substrate with template 8-oxodG or dG) was higher than the amount of products in the ligation assays (*i*.*e*., ligation of the nicked DNA substrate with preinserted 3′-rA or 3′-rC opposite template 8-oxodG or dG), as shown in the black bar graphs in Figs. 11–14. The degree of difference in the amount of ligation products varied depending on the type of template base or DNA ligase used in this study, as summarized in Supplementary Table 2. Overall comparisons indicate that the insertion of ribonucleotides (rATP or rCTP) into DNA by pol μ is necessary for the ligation step and thus NHEJ to occur efficiently *in vitro*. On the other hand, the results also suggest that differences in the ligation efficiency of ribonucleotide-containing repair intermediates could be due to the distinct sugar and mismatch discrimination mechanisms of pol µ and DNA ligase, respectively, depending on the architecture of the DNA ends during NHEJ.

## Discussion

The pool imbalance between the intracellular concentrations of rNTPs and dNTPs makes it challenging for RNA and DNA polymerases to select their correct sugar substrates^1,2^. Since the sugar-phosphate backbone of RNA is much more prone to strand breakage than that of DNA, potentially resulting in the accumulation of strand breaks, ribonucleotide insertion by DNA polymerases during replication and repair can constitute a major threat to genome stability^3,4^. The family X DNA polymerases, pol β, λ and μ, can incorporate rNTPs during the BER and NHEJ repair pathways with varying sugar selectivity^10,11^. These polymerases with no proofreading activity have also been shown to function in the translesion synthesis of oxidative DNA lesions such as 8-oxodG in an error-prone manner^15–20^. As a major oxidative base modification in DNA, 8-oxodG has a characteristic known as dual coding potential (in either the *anti* or *syn* conformation) that enables this base to form a correct base pairing with cytosine or a mispairing with adenine^45^. NHEJ, including several components of the repair machinery such as pol μ and the DNA ligase IV/XRCC4 complex, encounters this lesion in clusters at DNA strand break termini; however, the impact of pol μ ribonucleotide insertion during the translesion synthesis of 8-oxodG on the downstream ligation step of the repair pathway is unknown. Mn^2+^, as a mutagenic metal ion, is known to reduce DNA polymerase fidelity, and pol μ has a strong preference for Mn^2+^ over Mg^2+^ for its various activities, such as ribonucleotide insertion^21–24^. Moreover, it has been shown that physiological concentrations of Mn^2+^ and ribonucleotides enhance NHEJ with the cost of increased ribonucleotide insertion^25^. However, much less is known about the impact of the mutagenic metal ion on pol μ ribonucleotide insertion coupled with ligation during the NHEJ pathway.

We therefore investigated the ligation efficiency of pol μ ribonucleotide (rATP or rCTP) insertion products in the presence of a divalent ion (Mg^2+^ or Mn^2+^) using a model NHEJ repair substrate with a template 8-oxodG in the coupled double-strand break repair catalyzed by pol μ and DNA ligase and compared it with that of the ligation reaction by DNA ligase alone *in vitro*. Our results indicated that pol μ stabilizes the insertion products of ribonucleotides (rATP or rCTP) over the insertion products of deoxyribonucleotides (dATP or dCTP) opposite 8-oxodG or dG, allowing their efficient channeling to the next ligation step, which enables DNA ligase to function on the resulting nicked repair intermediate. On the other hand, the DNA ligases tested in this study (DNA ligase I and DNA ligase IV/XRCC4 complex) are compromised by noncanonical base pairings at the 3′-ends of the repair intermediates. The difference in our findings between a coupled reaction including pol µ and DNA ligase and the ligation reaction including DNA ligase alone (Figs. 11–14) revealed that the presence of ribonucleotides inserted by pol µ bypassing 8-oxodG is required for the efficient ligation of the repair intermediates *in vitro*. Our results demonstrate that pol µ ribonucleotide insertion during the translesion synthesis of 8-oxodG coupled with ligation could be a promutagenic event leading to genomic instability by NHEJ (Supplementary Model 1). Further research, that is, structure/function studies, will be necessary to enhance our understanding of the role of pol µ in the mutagenic potential of ribonucleotides embedded in genomic DNA. Similarly, *in vivo* studies will be required to understand the biological consequences of ribonucleotide incorporation in the oxidative stress-induced damage response during NHEJ in cells.

## Materials and Methods

### Materials

Oligodeoxyribonucleotides with and without a 6-carboxyfluorescein (FAM) label were obtained from Integrated DNA Technologies. Single-nucleotide gapped and nicked DNA substrates (Supplementary Table 1) were prepared as described previously^35,46^. The deoxyribo- and ribonucleoside triphosphate solutions (dATP or rATP and dCTP or rCTP) were obtained from GE Healthcare.

### Protein purifications

Human DNA polymerases, namely, full-length (Met1-Ala494) pol μ, the catalytic domain (Pro132-Ala494) of pol μ, and full-length pol β, were purified as described^35,46–48^. Briefly, the proteins were overexpressed in Rosetta2 (DE3) cells overnight at 16 °C. The cells were sonicated and lysed at 4 °C in lysis buffer containing 25 mM Tris-HCl (pH 8.0), 500 mM NaCl, 5% glycerol, 1 mM DTT, and cOmplete Protease Inhibitor Cocktail (Roche). The cell lysates were then clarified by centrifugation. The soluble proteins were bound to glutathione 4B sepharose resin (GE Healthcare) at 4 °C, and the polymerases were eluted by TEV cleavage overnight at 4 °C. The proteins were then purified by size exclusion chromatography (Superdex 200 16/60) followed by ion exchange (MonoQ HR 5/5) chromatography (GE Healthcare). The final proteins were dialyzed and concentrated in buffer containing 25 mM Tris-HCl (pH 8:0), 100 mM NaCl, 5% glycerol, and 1 mM DTT. Recombinant full-length human DNA ligase I was purified as previously described^36,38,47^. Briefly, the protein was expressed in Rosetta2 (DE3) cells at 37 °C, and the cells were grown overnight at 16 °C. After cell lysis by sonication at 4 °C in lysis buffer containing 40 mM HEPES (pH 7.5), 200 mM NaCl, 10% glycerol, and cOmplete Protease Inhibitor Cocktail (Roche) and clarification by centrifugation, the His-tagged protein was loaded onto a HisTrap HP column (GE Healthcare) and purified by elution with an increasing imidazole gradient (0–500 mM) at 4 °C, then subsequently loaded onto a HiTrap Q HP column (GE Healthcare) and eluted with NaCl. For all purified proteins used in this study, the final enzyme samples were concentrated, frozen in dry ice, and stored in aliquots at −80 °C.

### Coupled reaction assay

The repair assays that enable the measurement of deoxyribonucleotide or ribonucleotide insertion coupled with ligation (Supplementary Scheme 1) were performed under steady-state conditions *in vitro* as described previously^35,46^. The single-nucleotide gapped DNA substrates with a template 8-oxodG or dG are presented in Supplementary Table 1. The reaction mixture contained 50 mM Tris-HCl (pH 7.5), 1 mM DTT, 1 mM ATP, 100 µgml^−1^ BSA, the single-nucleotide gapped DNA substrate (500 nM), 100 µM rNTP (rATP or rCTP) or dNTP (dATP or dCTP), and MgCl_2_ (10 mM) or MnCl_2_ (1 mM) in a final volume of 10 µl. The reaction was initiated by the addition of the preincubated enzyme mixture, including DNA polymerase (pol μ or pol β, 100 nM) and DNA ligase (DNA ligase I or DNA ligase IV/XRCC4 complex, 100 nM). The reaction mixtures were then incubated at 37 °C for the times indicated in the figure legends. The reaction products were mixed with an equal amount of gel loading buffer (95% formamide, 20 mM EDTA, 0.02% bromophenol blue, and 0.02% xylene cyanol) and then separated by electrophoresis on an 18% polyacrylamide gel as described previously^35,46^. The gels were scanned with a Typhoon PhosphorImager (Amersham Typhoon RGB), and the data were analyzed with ImageQuant software. The control coupled reactions for pol μ correct base insertions (dATP:dT and dCTP:dG) were performed as described above.

### Nucleotide insertion assay

The nucleotide insertion assays (Supplementary Scheme 2) were performed under steady-state conditions *in vitro* as described previously^35,46^. The single-nucleotide gapped DNA substrates with a template 8-oxodG or dG are presented in Supplementary Table 1. The reaction mixture contained 50 mM Tris-HCl (pH 7.5), 1 mM DTT, 1 mM ATP, 100 µgml^−1^ BSA, the single-nucleotide gapped DNA substrate (500 nM), 100 µM rNTP (rATP or rCTP) and MgCl_2_ (10 mM) or MnCl_2_ (1 mM) in a final volume of 10 µl. The reaction was initiated by the addition of pol μ, and the reaction mixtures were then incubated at 37 °C for the time points indicated in the figure legends. The reaction products were mixed with an equal amount of gel loading buffer (95% formamide, 20 mM EDTA, 0.02% bromophenol blue, and 0.02% xylene cyanol) and then separated and analyzed as described above.

### Ligation assay

The ligation assays (Supplementary Scheme 3) were performed under steady-state conditions *in vitro* as described previously^35,46^. The nicked DNA substrates with 3′-preinserted ribonucleotides (3′-rA or 3′-rC) opposite 8-oxodG or dG are presented in Supplementary Table 1. The reaction mixture contained 50 mM Tris-HCl (pH 7.5), 1 mM DTT, 1 mM ATP, 100 µgml^−1^ BSA, the nicked DNA substrate (500 nM) and MgCl_2_ (10 mM) or MnCl_2_ (1 mM) in a final volume of 10 µl. The experiments were initiated with the addition of DNA ligase (DNA ligase I or DNA ligase IV/XRCC4 complex, 100 nM). The reaction mixture was incubated at 37 °C until the time points indicated in the figure legends and then stopped by mixing with an equal volume of loading dye. The reaction products were analyzed as described above. Control ligation assays with nicked DNA substrates, including correct base pairs (3′-dA:dT and 3′-dC:dG), were performed as described above.

## Supplementary information

Supplementary information.

## References

[1] Klein HL (2017). Genome instabilities arising from ribonucleotides in DNA. DNA Repair.

[2] Potenski CJ, Klein HL (2014). How the misincorporation of ribonucleotides into genomic DNA can be both harmful and helpful to cells. Nuc. Acids Res..

[3] Sassa A, Yasui M, Honma M (2019). Current perspectives on mechanisms of ribonucleotide incorporation and processing in mammalian DNA. Genes Environ..

[4] Yao NY, Schroeder JW, Yurieva O, Simmons LA, O’Donnell ME (2013). Cost of rNTP/dNTP pool imbalance at the replication fork. Proc. Natl. Acad. Sci..

[5] Khanna KK, Jackson SP (2001). DNA double-strand breaks: signaling, repair and the cancer connection. Nat. Genet..

[6] Ma Y, Lu H, Schwarz K, Lieber MR (2005). Repair of double-strand DNA breaks by the human nonhomologous DNA end joining pathway: the iterative processing model. Cell Cycle.

[7] Chayot R, Montagne B, Ricchetti M (2012). DNA polymerase μ is a global player in the repair of non-homologous end-joining substrates. DNA Repair.

[8] McElhinny SA, Ramsden DA (2003). Polymerase μ is a DNA-directed DNA/RNA polymerase. Mol. Cell Biol..

[9] Chayot R, Danckaert A, Montagne B, Ricchetti M (2010). Lack of DNA polymerase μ affects the kinetics of DNA double-strand break repair and impacts on cellular senescence. DNA Repair.

[10] Brown JA (2010). A novel mechanism of sugar selection utilized by a human X-family DNA polymerase. J. Mol. Biol..

[11] Brown JA, Suo Z (2011). Unlocking the sugar ‘steric gate’ of DNA polymerases. Biochemistry.

[12] Moon AF (2017). Structural accommodation of ribonucleotide incorporation by the DNA repair enzyme polymerase μ. Nuc. Acids Res..

[13] Ruiz JF (2014). Lack of sugar discrimination by human pol μ requires a single glycine residue. Nuc. Acids Res..

[14] Moon AF (2014). Sustained active site rigidity during synthesis by human DNA polymerase μ. Nat. Struct. Mol. Biol..

[15] Covo S, Blanco L, Livneh Z (2004). Lesion bypass by human DNA polymerase μ reveals a template-dependent, sequence-independent nucleotidyl transferase activity. J. Biol. Chem..

[16] Dominguez O (2000). DNA polymerase μ, homologous to TdT, could act as a DNA mutator in eukaryotic cells. EMBO J..

[17] Havener JM (2003). Translesion synthesis past platinum DNA adducts by human DNA polymerase μ. Biochemistry.

[18] Ruiz JF (2001). DNA polymerase μ, a candidate hypermutase?. Philos. Trans. R. Soc. Lond. B. Biol. Sci..

[19] Zhang Y (2002). Lesion bypass activities of human DNA polymerase μ. J. Biol. Chem..

[20] Kaminski AM (2019). Unexpected behavior of DNA polymerase μ opposite template 8-oxo-7,8-dihydro-2′-guanosine. Nuc. Acids Res..

[21] Sirover MA, Loeb LA (1976). Infidelity of DNA synthesis *in vitro*: screening for potential metal mutagens or carcinogens. Science.

[22] Goodman MF, Keener S, Guidotti S, Branscomb EW (1983). On the enzymatic basis for mutagenesis by manganese. J. Biol. Chem..

[23] Beckman RA, Mildvan AS, Loeb LA (1985). On the fidelity of DNA replication: manganese mutagenesis *in vitro*. Biochemistry.

[24] Hays H, Berdis AJ (2002). Manganese substantially alters the dynamics of translesion DNA synthesis. Biochemistry.

[25] Martin MJ, Ortiz-Garcia MV, Esteban V, Blanco L (2013). Ribonucleotides and manganese ions improve non-homologous end joining by human pol μ. Nuc. Acids Res..

[26] Chang YK (2019). Human DNA polymerase μ can use a nancanonical mechanism for multiple Mn^2+^-mediated functions. J. Am. Chem. Soc..

[27] Waters CA, Strande NT, Wyatt DW, Pryor JM, Ramsden DA (2014). Nonhomologous end joining: a good solution for bad ends. DNA Repair.

[28] Lieber MR (2008). The mechanism of human nonhomologous DNA end joining. J. Biol. Chem..

[29] Martin MJ, Blanco L (2014). Decision-making during NHEJ: a network of interactions in human pol μ implicated in substrate recognition and end-bridging. Nuc. Acids Res..

[30] Kuhfittig-Kulle S (2007). The mutagenic potential of non-homologous end joining in the absence of the NHEJ core factors Ku70/80, DNA-PKcs and XRCC4-LigIV. Mutagenesis.

[31] Davis B, Havener JM, Ramsden DA (2008). End-bridging is required for pol μ to efficiently promote repair of noncomplementary ends by nonhomologous end joining. Nuc. Acids Res..

[32] Mahajan KN, McElhinny SA, Mitchell BS, Ramsden DA (2002). Association of DNA polymerase μ with Ku and Ligase IV: Role for pol μ in end-joining double-strand break repair. Mol. Cell. Biol..

[33] Waters CA (2014). The fidelity of the ligation step determines how ends are resolved during nonhomologous end joining. Nat. Commun..

[34] Conlin MP (2017). DNA Ligase IV guides end-processing choice during nonhomologous end joining. Cell Rep..

[35] Çağlayan M, Wilson SH (2018). Pol μ dGTP mismatch insertion opposite T coupled with ligation reveals a promutagenic DNA intermediate during double strand break repair. Nat. Commun..

[36] Çağlayan M, Wilson SH (2015). Oxidant and environmental toxicant-induced effects compromise DNA ligation during base excision DNA repair. DNA Repair.

[37] Çağlayan M (2019). Interplay between DNA polymerases and DNA ligases: Influence on substrate channeling and the fidelity of DNA ligation. J. Mol. Biol..

[38] Pryor JM (2018). Ribonucleotide incorporation enables repair of chromosome breaks by nonhomologous end joining. Science.

[39] Ramden DA (2011). Polymerases in nonhomologous end joining: Building a bridge over broken chromosomes. Antioxid. Redox Signal.

[40] Tseng HM, Tomkinson AE (2002). A physical and functional interaction between yeast Pol4 and Dnl4-Lif1 links DNA synthesis and ligation in nonhomologous end joining. J. Biol. Chem..

[41] Crespan E (2016). Impact of ribonucleotide incorporation by DNA polymerases β and λ on oxidative base excision repair. Nat. Comm..

[42] Cavanaugh NA, Beard WA, Wilson SH (2010). DNA polymerase β ribonucleotide discrimination. J. Biol. Chem..

[43] Pelletier H, Sawaya MR, Wolfle W, Wilson SH, Kraut J (1996). A Structural basis for metal ion mutagenicity and nucleotide selectivity in human DNA polymerase β. Biochemistry.

[44] Ray S (2018). DNA polymerase beta participates in DNA end-joining. Nucleic Acids Res..

[45] Kamiya H (2003). Mutagenic potentials of damaged nucleic acids produced by reactive oxygen/nitrogen species: approaches using synthetic oligonucleotides and nucleotides: survey and summary. Nucleic Acids Res..

[46] Çağlayan M, Horton JK, Stefanick DF, Wilson SH (2017). Oxidized nucleotide insertion by pol β confounds ligation during base excision repair. Nat. Commun..

[47] Beard WA, Wilson SH (1995). Purification and domain-mapping of mammalian DNA polymerase beta. Methods in Enzymology.

[48] Howes TRL (2017). Structure-activity relationships among DNA ligase inhibitors: Characterization of a selective uncompetitive DNA ligase I inhibitor. DNA Repair.

