# Supplementary material for: Pol μ ribonucleotide insertion opposite 8-oxodG facilitates the ligation of premutagenic DNA repair intermediate
Source: Sci Rep. 2020 Jan 22;10:940. doi: 10.1038/s41598-020-57886-y (PMC6976671; doi:10.1038/s41598-020-57886-y)
Supplement: Supplementary file 1 — Supplementary information. [file 41598_2020_57886_MOESM1_ESM.pdf]

Supplementary Information

Pol μ ribonucleotide insertion opposite 8-oxodG facilitates the ligation of premutagenic DNA repair intermediate

Melike Çağlayan

Department of Biochemistry and Molecular Biology, University of Florida, Gainesville, FL 32610, USA

* To whom correspondence should be addressed. Tel: 352-2948383; Fax: 352-3922953; Email: caglayanm@ufl.edu

Supplementary Figures 1-24

Supplementary Tables 1 and 2

Supplementary Schemes 1-3

Supplementary Model 1

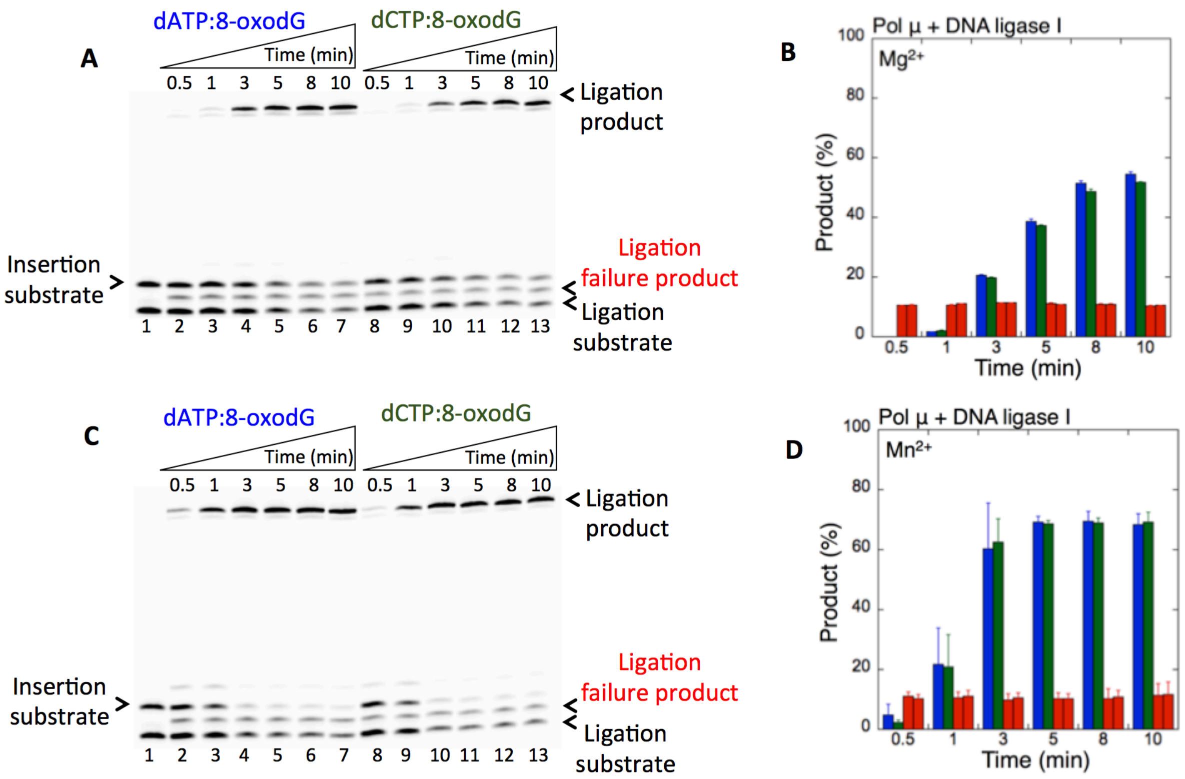

Supplementary Figure 1. Pol µ dATP and dCTP insertion opposite 8-oxodG coupled with ligation by DNA ligase I in the presence of Mg^{2+} vs Mn^{2+}. (**A,C**) In both panels, lane 1 is the minus enzyme control for the one nucleotide gapped DNA substrate with template 8-oxodG. Lanes 2-7 and 8-13 are the coupled reaction products in the presence of dATP or dCTP, respectively, and correspond to time points of 0.5, 1, 3, 5, 8, and 10 min. (**B,D**) Graphs show time-dependent changes in the products of ligation (blue and green) and ligation failure (red) in the presence of Mg^{2+} (B) and Mn^{2+} (D). The data represent the average of three independent experiments ± SD.

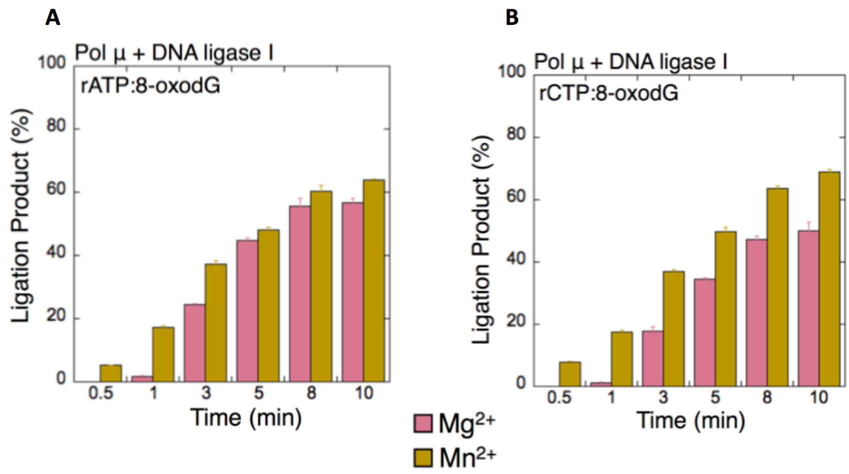

Supplementary Figure 2. Graphs show the comparisons for the time-dependent changes in the products of ligation by pol μ and DNA ligase I for rATP:8-oxodG (**A**) and rCTP:8-oxodG (**B**) in the presence of Mg^{2+} vs Mn^{2+}. The data represent the average of three independent experiments ± SD.

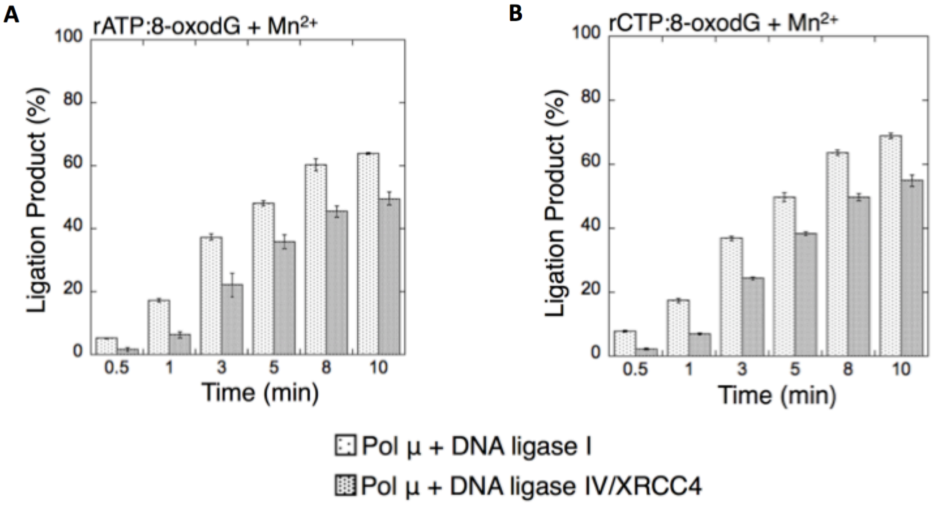

Supplementary Figure 3. Graphs show the comparisons for the time-dependent changes in the products of ligation by pol μ and DNA ligase I vs pol μ and DNA ligase IV/XRCC4 complex in the presence of Mn^{2+} for rATP:8-oxodG (**A**) and rCTP:8-oxodG (**B**). The data represent the average of three independent experiments ± SD.

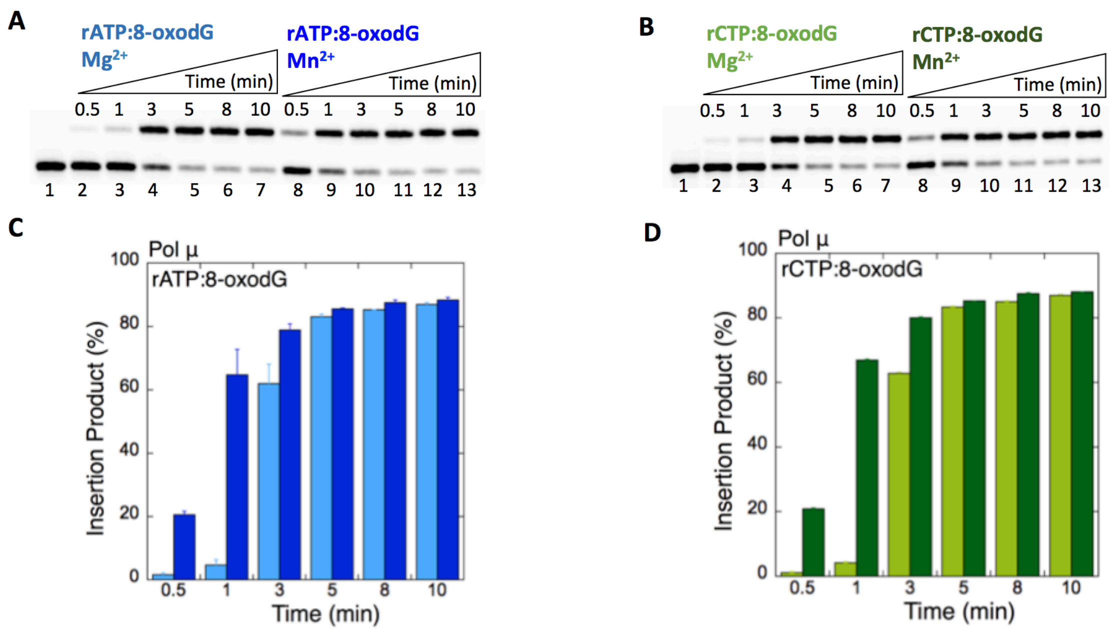

Supplementary Figure 4. Pol μ rATP and rCTP insertion opposite 8-oxodG in the presence of Mg^{2+} vs Mn^{2+}. (**A,B**) In both panels, lane 1 is the minus enzyme control for the one nucleotide gapped DNA substrate with template 8-oxodG. Lanes 2-7 and 8-13 are the insertion reaction products for rATP:8-oxodG (A) and rCTP:8-oxodG (B) in the presence of Mg^{2+} vs Mn^{2+}, respectively, and correspond to time points of 0.5, 1, 3, 5, 8, and 10 min. (**C,D**) Graphs show time-dependent changes in the products of insertion. The data represent the average of three independent experiments ± SD.

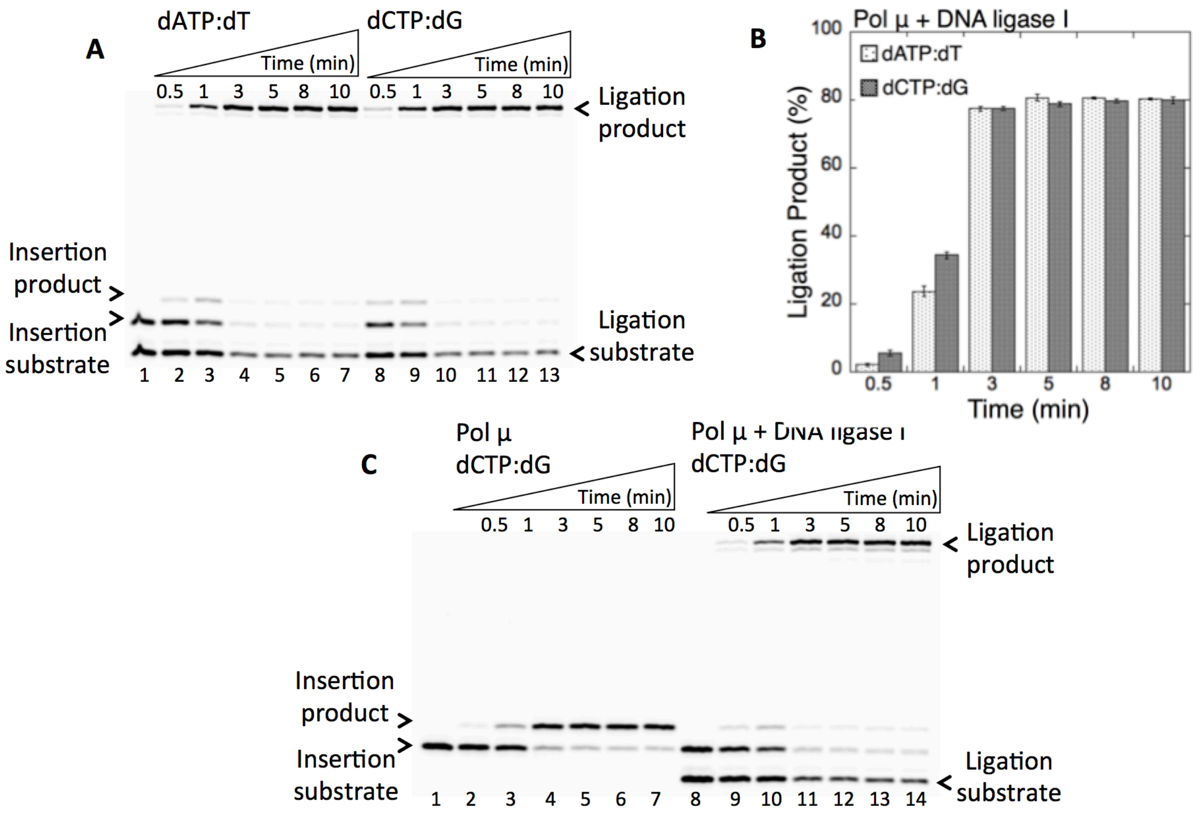

Supplementary Figure 5. Pol μ dATP:dT and dCTP:dG insertion coupled with ligation by DNA ligase I. (**A**) Lane 1 is the minus enzyme control for the one nucleotide gapped DNA substrate with template dT. Lanes 2-7 and 8-13 are the coupled reaction products, and correspond to time points of 0.5, 1, 3, 5, 8, and 10 min. (**B**) Graph shows time-dependent changes in the products of ligation and the data represent the average of three independent experiments ± SD. (**C**) Lanes 1 and 8 are the minus enzyme controls for the one nucleotide gapped DNA substrates with template dG fort he insertion and coupled reactions, respectively. Lanes 2-7 and 9-14 are the products of insertion and coupled reactions for dCTP:dG, respectively, and correspond to time points of 0.5, 1, 3, 5, 8, and 10 min.

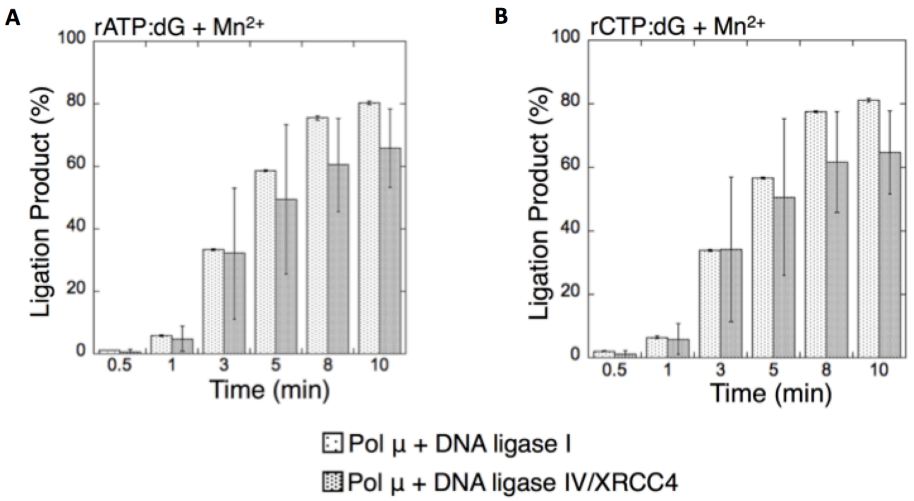

Supplementary Figure 6. Graphs show the comparisons for the time-dependent changes in the products of ligation by pol μ and DNA ligase I vs pol μ and DNA ligase IV/XRCC4 complex in the presence of Mn^{2+} for rATP:dG (**A**) and rCTP:dG (**B**). The data represent the average of three independent experiments ± SD.

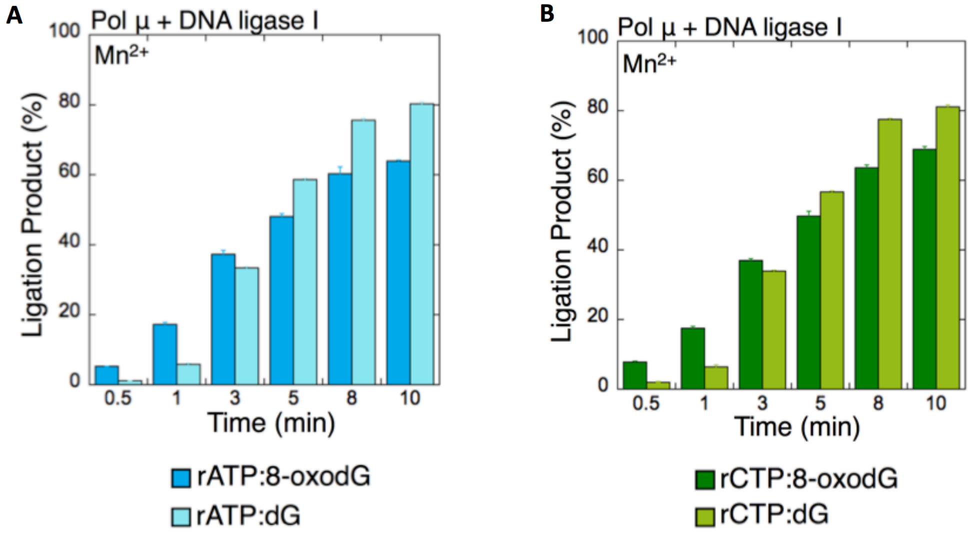

Supplementary Figure 7. Graphs show the comparisons for the time-dependent changes in the products of ligation by pol μ and DNA ligase I in the presence of Mn^{2+} for rATP:8-oxodG vs rATP:dG (**A**) and rCTP:8-oxodG vs rCTP:dG (**B**). The data represent the average of three independent experiments ± SD.

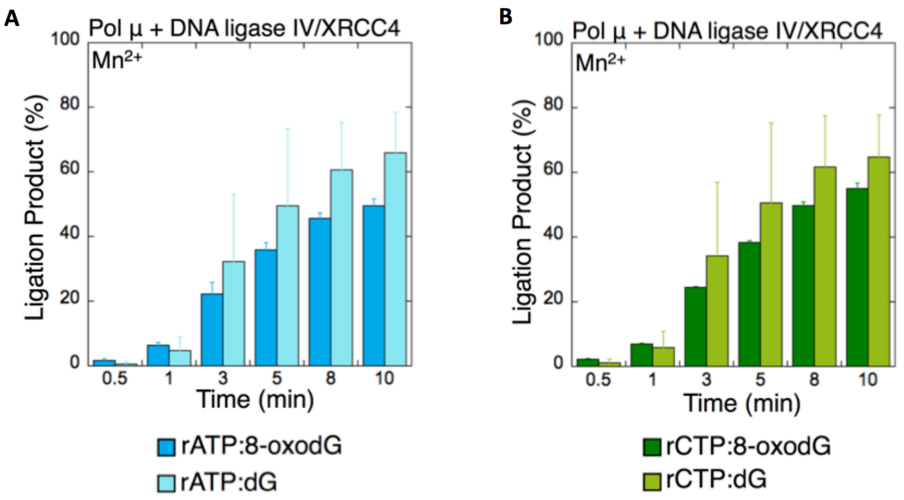

Supplementary Figure 8. Graphs show the comparisons for the time-dependent changes in the products of ligation by pol μ and DNA ligase IV/XRCC4 complex in the presence of Mn^{2+} for rATP:8-oxodG vs rATP:dG (**A**) and rCTP:8-oxodG vs rCTP:dG (**B**). The data represent the average of three independent experiments ± SD.

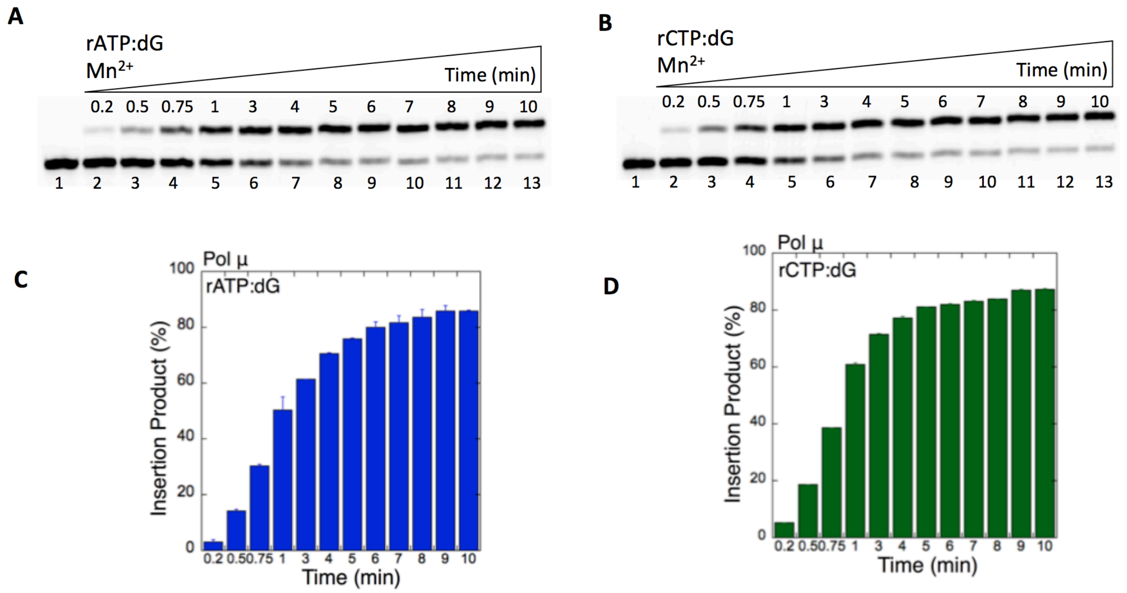

Supplementary Figure 9. Pol µ rATP and rCTP insertion opposite dG. (**A,B**) In both panels, lane 1 is the minus enzyme control for the one nucleotide gapped DNA substrate with template dG. Lanes 2-13 are the insertion reaction products for rATP:dG (A) and rCTP:dG (B), and correspond to time points of 0.2, 0.5, 0.75, 1, 3, 4, 5, 6, 7, 8, 9, and 10 min. (**C,D**) Graphs show time-dependent changes in the products of insertion (blue for rATP:dG and green for rCTP:dG). The data represent the average of three independent experiments ± SD.

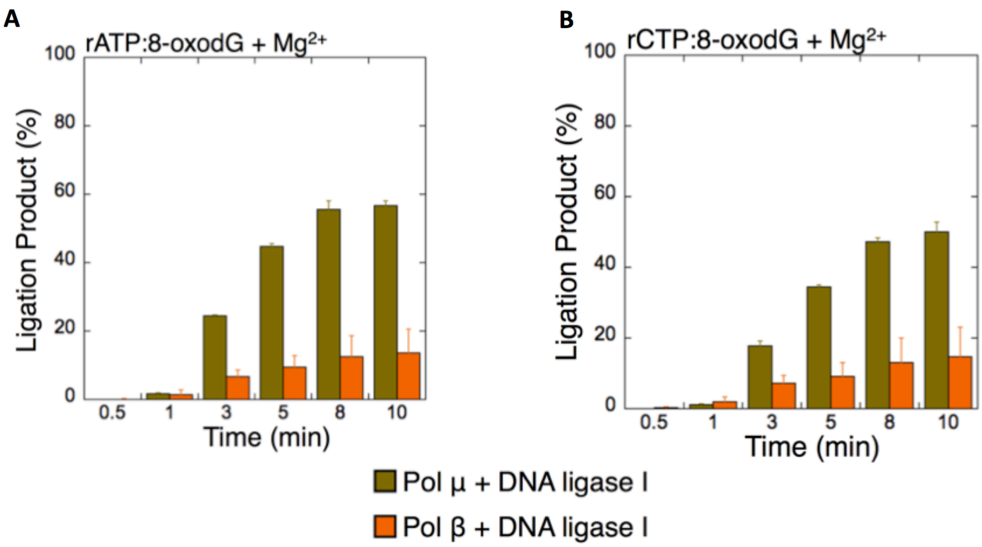

Supplementary Figure 10. Graphs show the comparisons for the time-dependent changes in the products of ligation by pol μ and DNA ligase I vs pol β and DNA ligase I in the presence of Mg^{2+} for rATP:8-oxodG (**A**) and rCTP:8-oxodG (**B**). The data represent the average of three independent experiments ± SD.

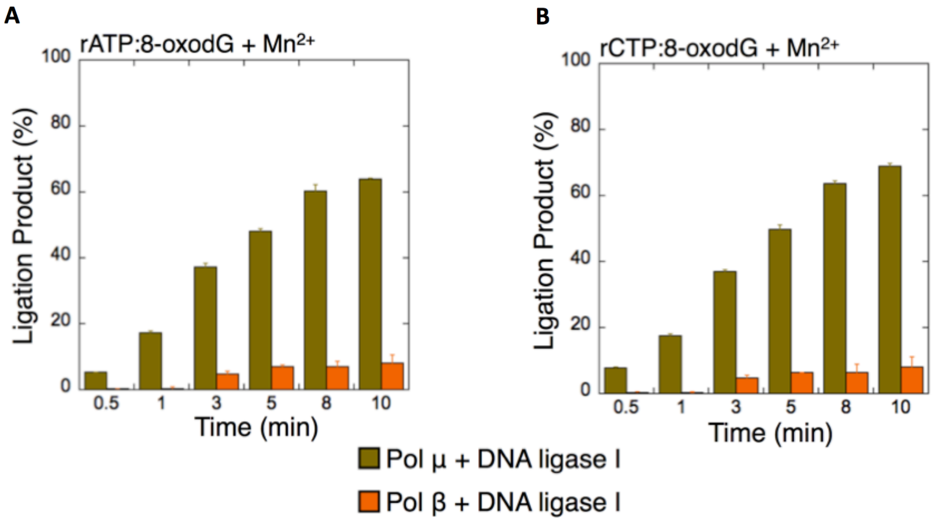

Supplementary Figure 11. Graphs show the comparisons for the time-dependent changes in the products of ligation by pol μ and DNA ligase I vs pol β and DNA ligase I in the presence of Mn^{2+} for rATP:8-oxodG (**A**) and rCTP:8-oxodG (**B**). The data represent the average of three independent experiments ± SD.

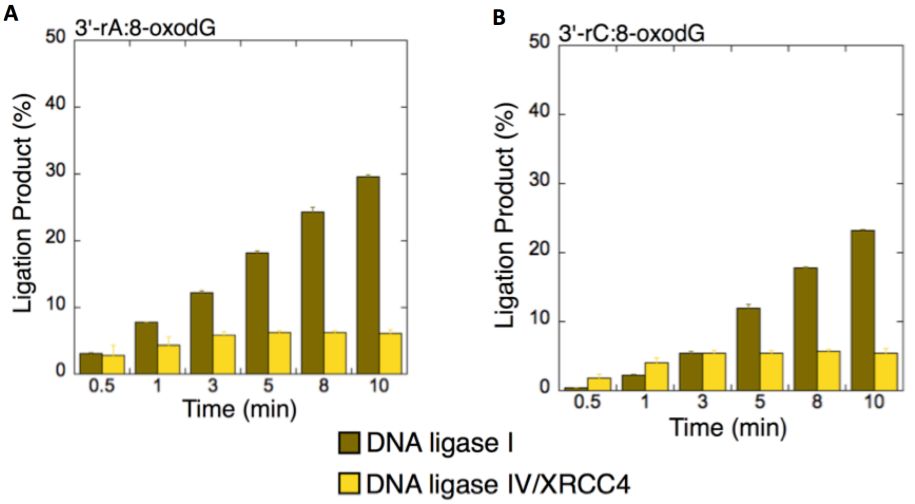

Supplementary Figure 12. Graphs show the comparisons for the time-dependent changes in the products of ligation by DNA ligase I vs DNA ligase IV/XRCC4 complex for 3'-rA:8-oxodG (**A**) and 3'-rC:8-oxodG (**B**). The data represent the average of three independent experiments ± SD.

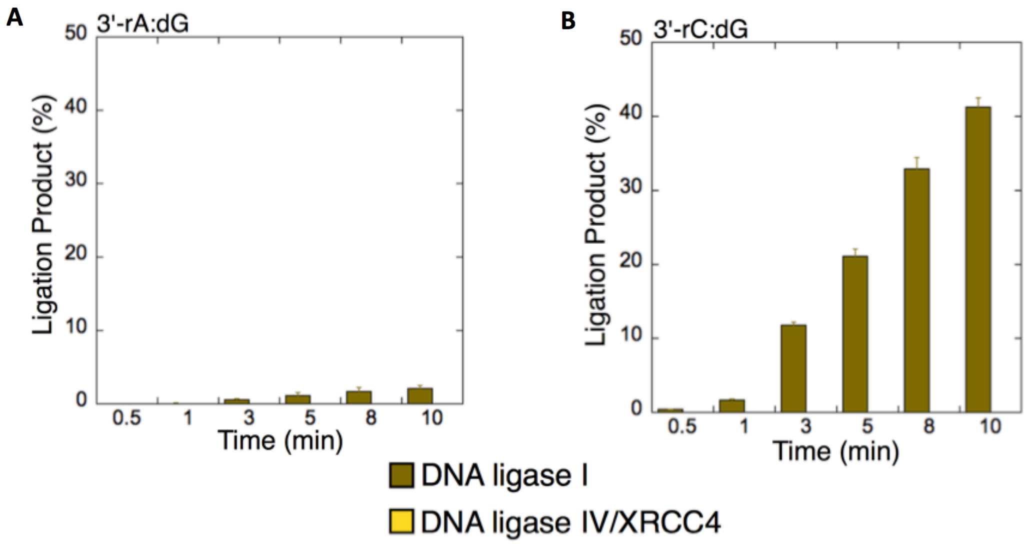

Supplementary Figure 13. Graphs show the comparisons for the time-dependent changes in the products of ligation by DNA ligase I vs DNA ligase IV/XRCC4 complex for 3'-rA:dG (**A**) and 3'-rC:dG (**B**). The data represent the average of three independent experiments ± SD.

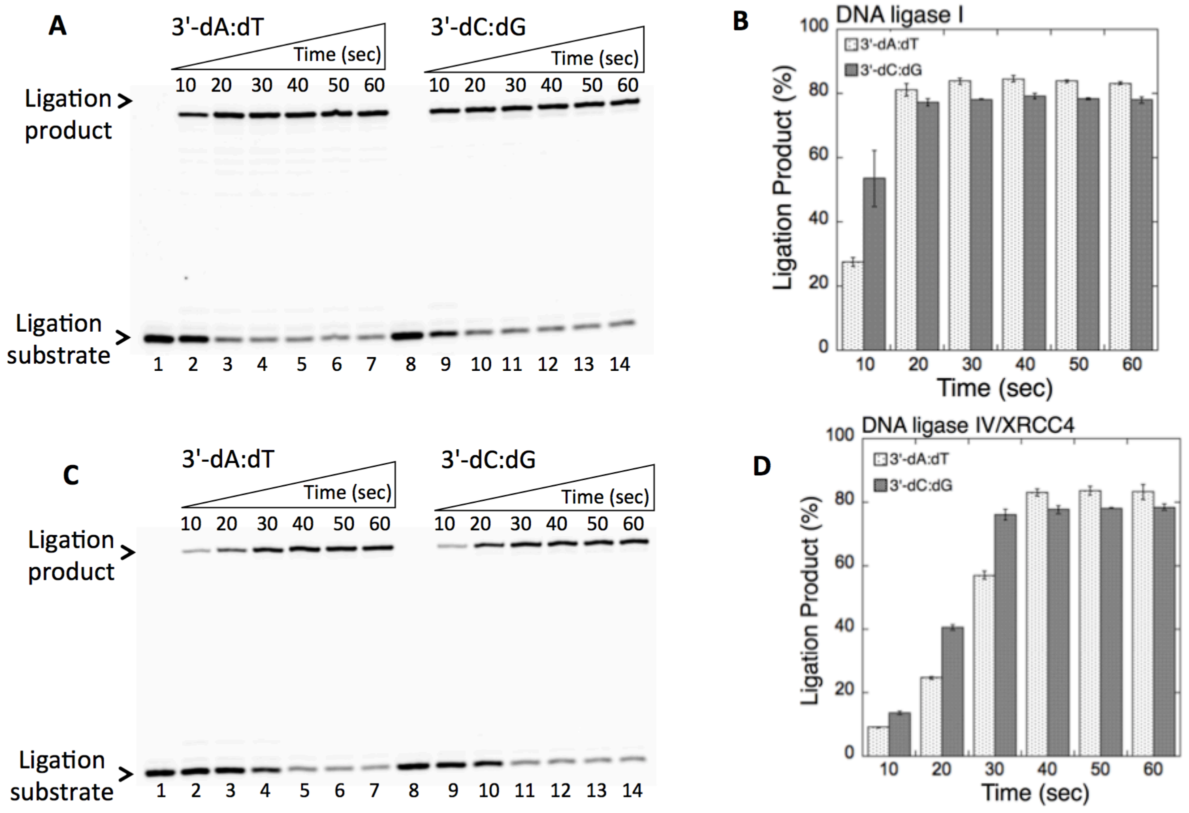

Supplementary Figure 14. Ligation of preinserted 3'-dA opposite dT and 3'-dC opposite dG by DNA ligase I vs DNA ligase IV/XRCC4 complex. (**A,C**) In both panels, lanes 1 and 8 are the minus enzyme controls for the nicked DNA substrates with 3'-dA:dT and 3'-dC:dG, respectively. Lanes 2-7 and 9-14 are the ligation products, and correspond to time points of 0.5, 1, 3, 5, 8, and 10 min. (**B,D**) Graphs show time-dependent changes in the products of ligation by DNA ligase I (B) and DNA ligase IV/XRCC4 complex (D). The data represent the average of three independent experiments ± SD.

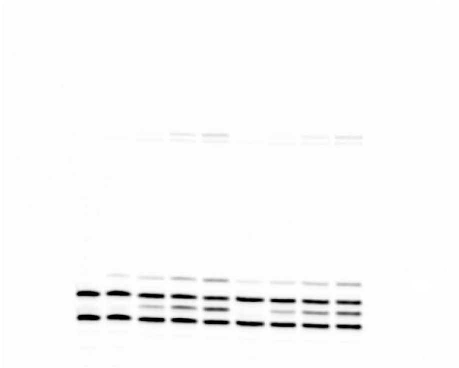

Supplementary Figure 15. Pol μ dATP and dCTP insertion opposite 8-oxodG coupled with ligation by DNA ligase I in the presence of Mg^{2+}.

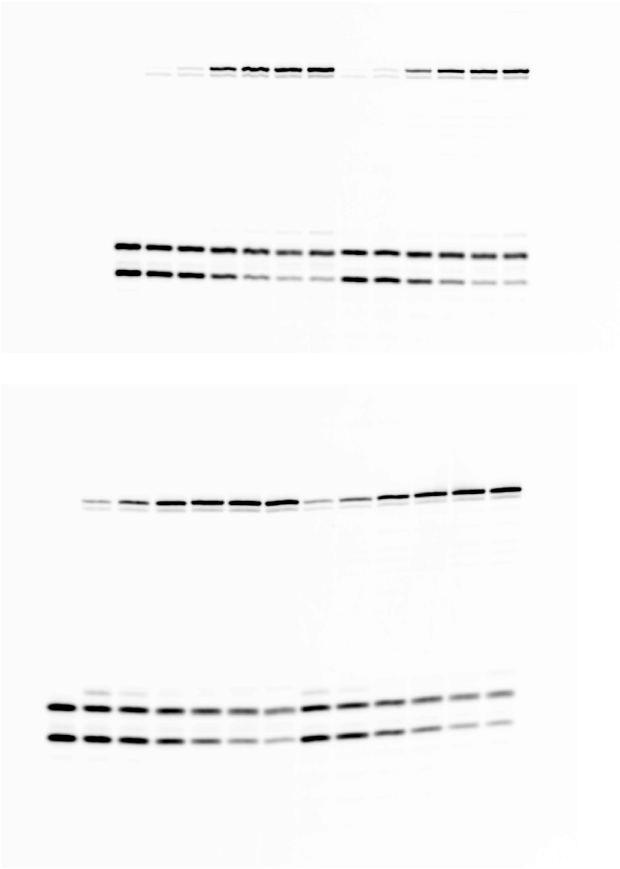

Supplementary Figure 16. Pol μ rATP and rCTP insertion opposite 8-oxodG coupled with ligation by DNA ligase I in the presence of Mg^{2+} vs Mn^{2+}.

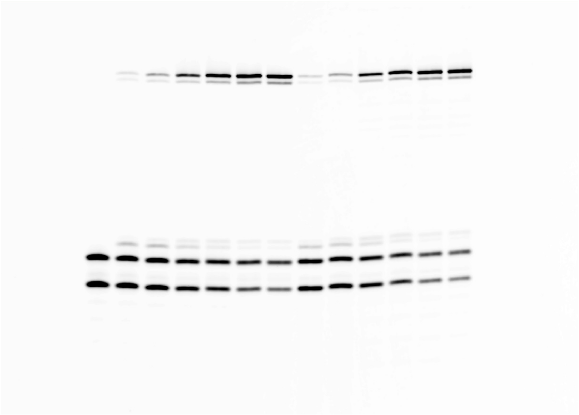

Supplementary Figure 17. Pol μ rATP and rCTP insertion opposite 8-oxodG coupled with ligation by DNA ligase IV/XRCC4 complex in the presence of Mn^{2+}.

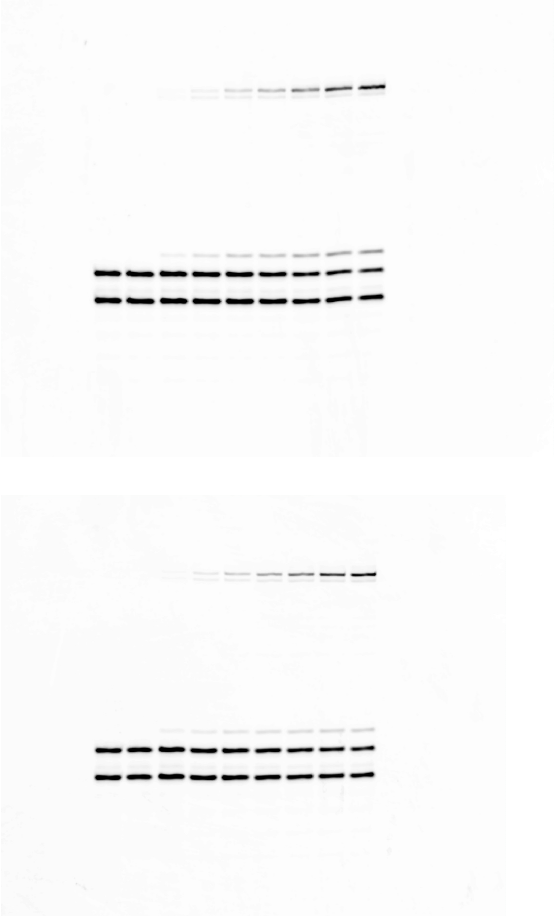

Supplementary Figure 18. Pol μ rATP and rCTP insertion opposite 8-oxodG coupled with ligation by DNA ligase IV/XRCC4 complex in the presence of Mg^{2+} vs Mn^{2+}.

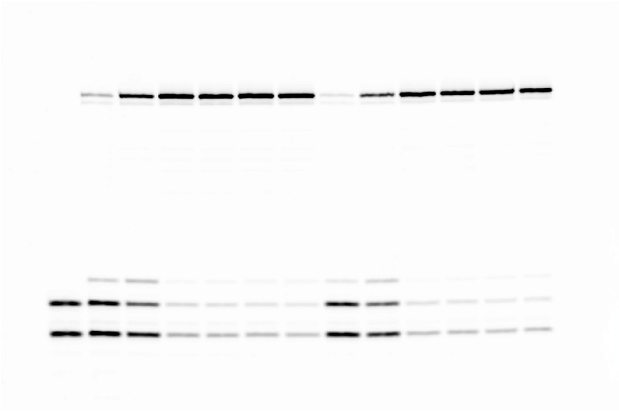

Supplementary Figure 19. Full-length pol μ rATP and rCTP insertion opposite 8-oxodG coupled with ligation by DNA ligase IV/XRCC4 complex.

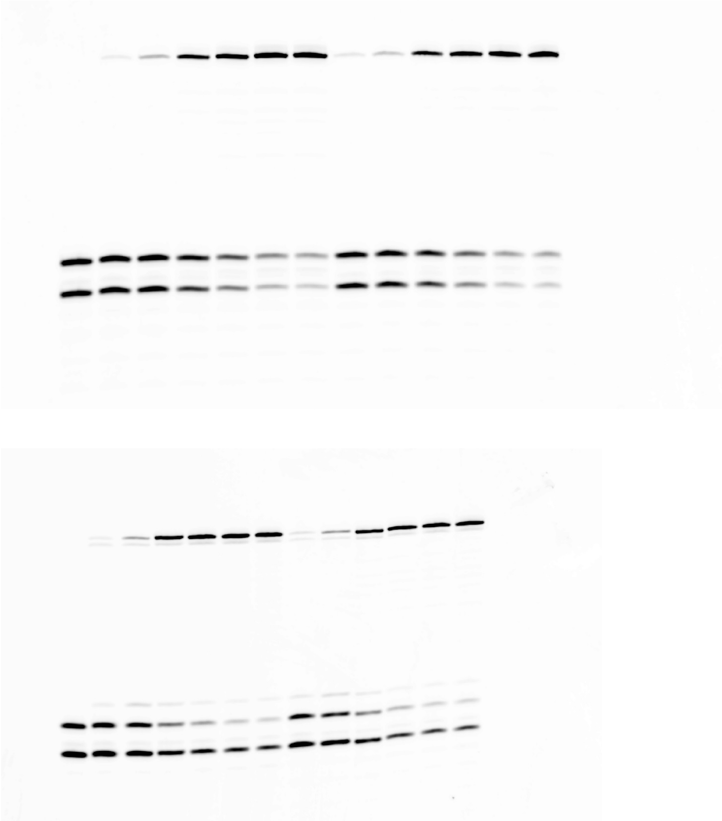

Supplementary Figure 20. Pol μ rATP and rCTP insertion opposite dG coupled with ligation by DNA ligase I vs DNA ligase IV/XRCC4 complex.

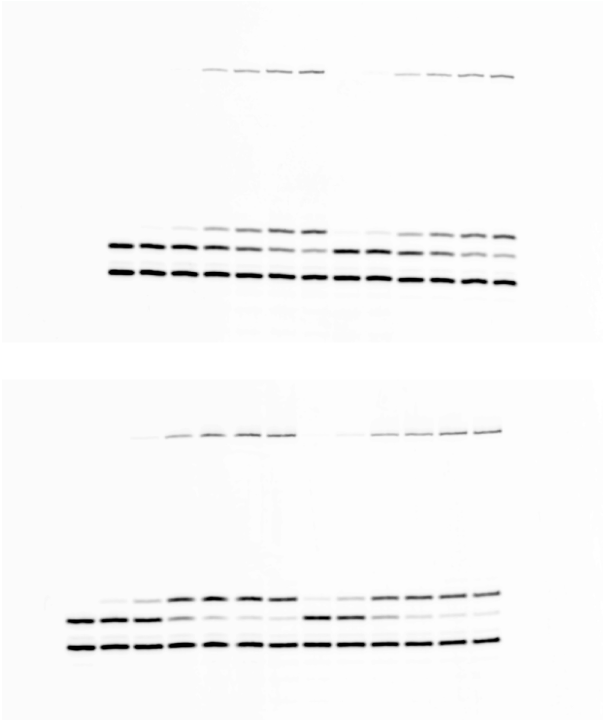

Supplementary Figure 21. Pol β rATP and rCTP insertion opposite 8-oxodG coupled with ligation by DNA ligase I in the presence of Mg^{2+} vs Mn^{2+}.

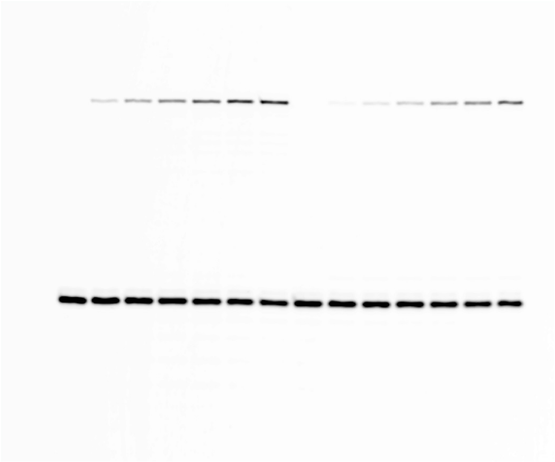

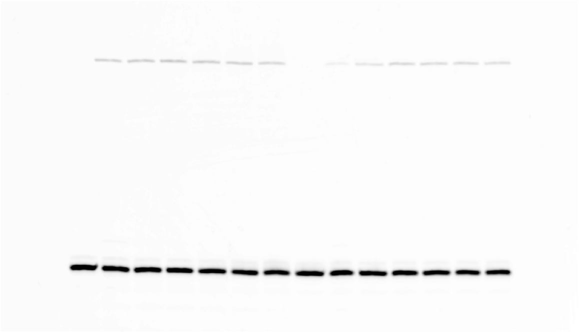

Supplementary Figure 22. The ligation of preinserted 3'-rA and 3'-rC opposite 8-oxodG by DNA ligase I vs DNA ligase IV/XRCC4 complex.

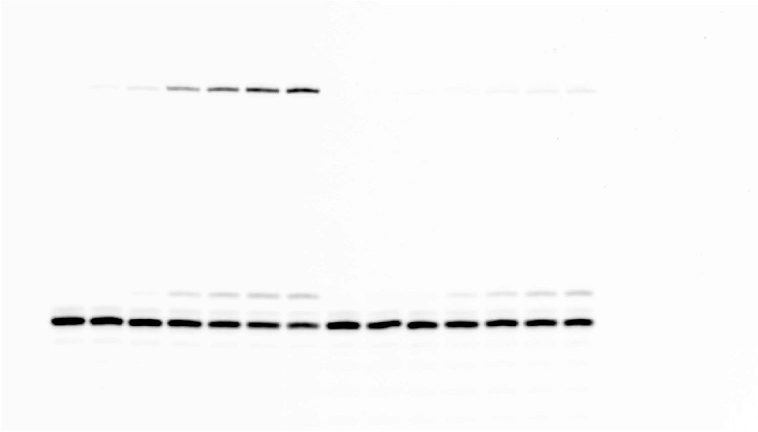

Supplementary Figure 23. The ligation of preinserted 3'-rC and 3'-rA opposite dG by DNA ligase I.

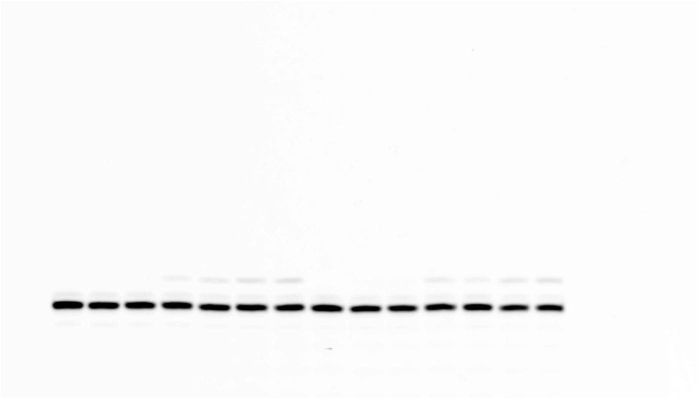

Supplementary Figure 24. The ligation of preinserted 3'-rC and 3'-rA opposite dG by DNA ligase IV/XRCC4 complex.

Substrate	Sequence
Substrate $X^{coupled}$	5'-FAM-CATGGGCGGCATGAACC GAGGCCCATCCTCACC-FAM-3'<br>3'-GTACCCGCCGTACTTGGXCTCCGGGTAGGAGTGG-5'
Substrate $G^{coupled}$	5'-FAM-CATGGGCGGCATGAACC GAGGCCCATCCTCACC-FAM-3'<br>3'-GTACCCGCCGTACTTGGGCTCCGGGTAGGAGTGG-5'
Substrate $X^{insertion}$	5'-FAM-CATGGGCGGCATGAACC GAGGCCCATCCTCACC-3'<br>3'-GTACCCGCCGTACTTGGXCTCCGGGTAGGAGTGG-5'
Substrate $G^{insertion}$	5'-FAM-CATGGGCGGCATGAACC GAGGCCCATCCTCACC-3'<br>3'-GTACCCGCCGTACTTGGGCTCCGGGTAGGAGTGG-5'
Substrate X^{nicked}	5'-CATGGGCGGCATGAACCNGAGGCCCATCCTCACC-FAM-3'<br>3'-GTACCCGCCGTACTTGGXCTCCGGGTAGGAGTGG-5'
Substrate G^{nicked}	5'-CATGGGCGGCATGAACCNGAGGCCCATCCTCACC-FAM-3'<br>3'-GTACCCGCCGTACTTGGGCTCCGGGTAGGAGTGG-5'
Substrate T^{nicked}	5'-CATGGGCGGCATGAACCNGAGGCCCATCCTCACC-FAM-3'<br>3'-GTACCCGCCGTACTTGGTCTCCGGGTAGGAGTGG-5'

Supplementary Table 1. The one nucleotide gapped and nicked DNA substrates used in this study. FAM indicates the presence of a fluorescence tag, N represents 3'-preinserted ribonucleotide (3'-rA and 3'-rC) or deoxyribonucleotide (3'-dA and 3'-dC) base, X is for 8-oxodG, and a base at the template base position is underlined.

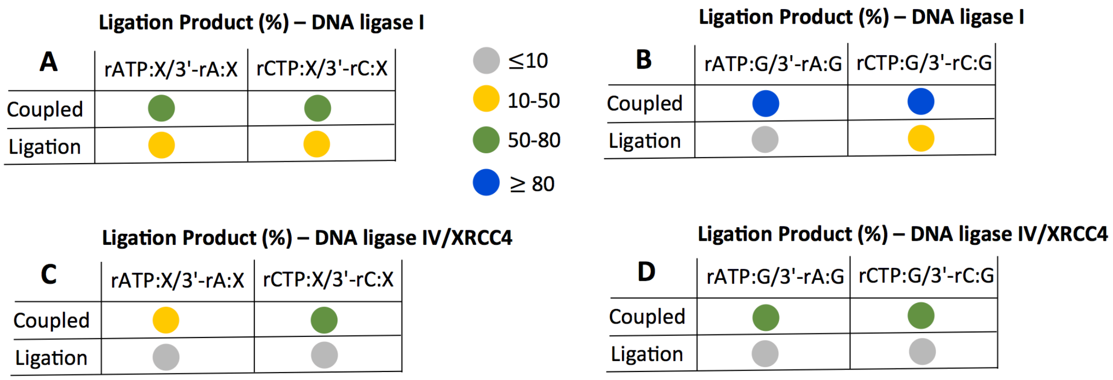

Supplementary Table 2. The comparison of the ligation products between the coupled reaction including pol μ and DNA ligase I or DNA ligase IV/XRCC4 vs the ligation reaction including DNA ligase I or DNA ligase IV/XRCC4. The data represent the percentage of ligation products for 10 min. The all time points (0.5-10 min) were presented as bar graphs in Figures 11-14.

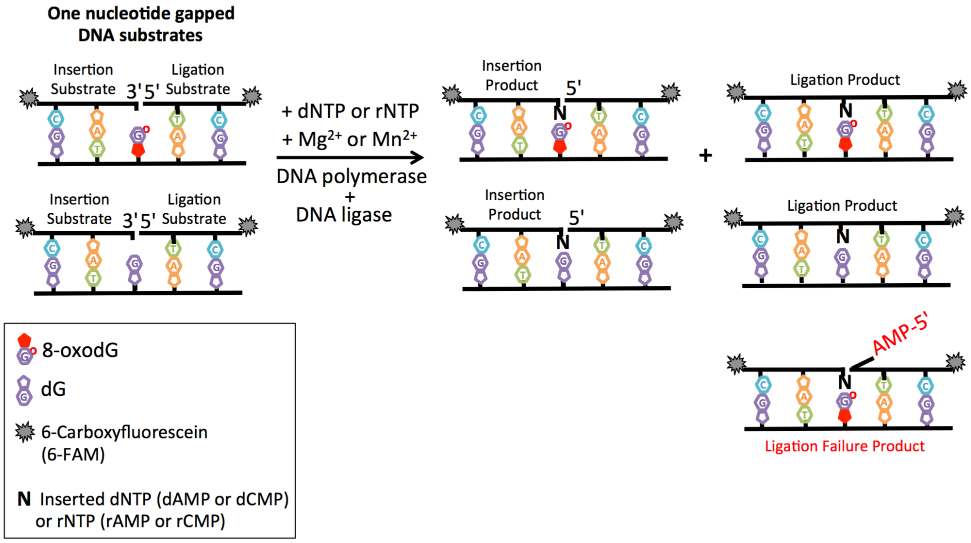

Supplementary Scheme 1. Ilustration of the coupled assay that enables to measure DNA polymerase (pol μ or pol β) dNTP (dATP or dCTP) or rNTP (rATP or rCTP) insertion coupled with ligation by DNA ligase (DNA ligase I or DNA ligase IV/XRCC4) in the presence of Mg^{2+} or Mn^{2+}. The single-nucleotide gapped DNA substrate includes a template base (8-oxodG or dG) and two fluorescent (FAM) labels at both 5'- and 3'-ends.

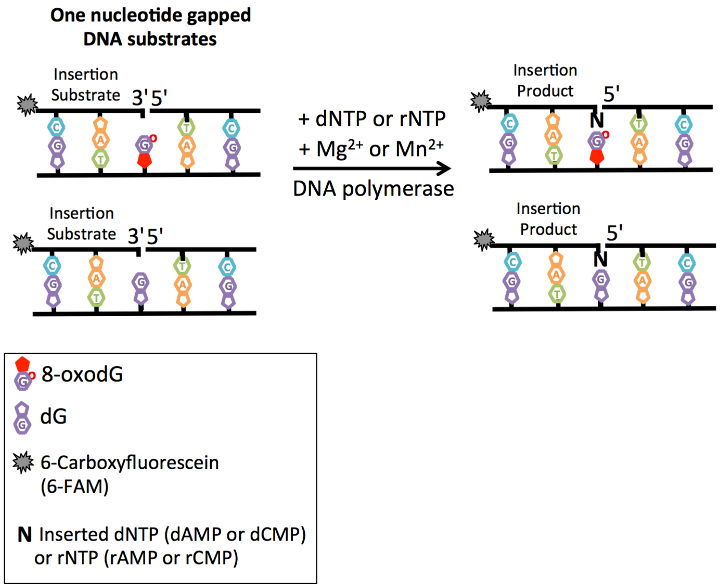

Supplementary Scheme 2. Ilustration of the nucleotide insertion assay that enables to measure dNTP (dATP or dCTP) or rNTP (rATP or rCTP) insertion by DNA polymerase (pol μ or pol β) alone. The single-nucleotide gapped DNA substrate includes a template base (8-oxodG or dG) and a fluorescent (FAM) label at 5'-end.

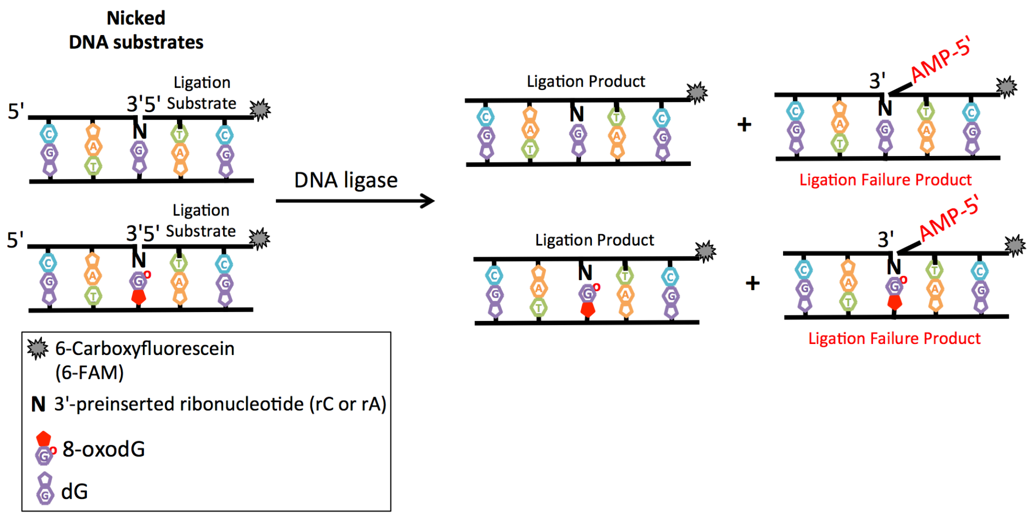

Supplementary Scheme 3. Ilustration of the ligation assay that enables to measure the ligation by DNA ligase (DNA ligase I or DNA ligase IV/XRCC4 complex) alone. The nicked DNA substrate contains a preinserted ribonucleotide (3'-rA and 3'-rC) or deoxyribonucleotide (3'-dA and 3'-dC) base, a template base (8-oxodG or dG), and a fluorescent (FAM) label at 3'-end.

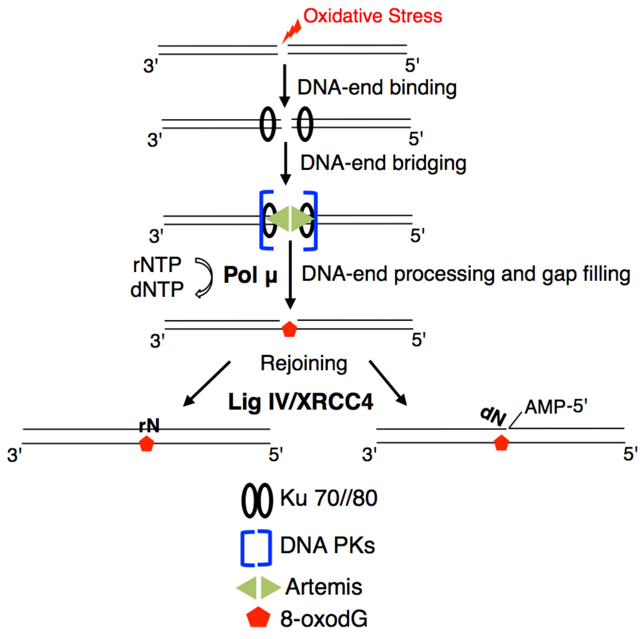

Supplementary Model 1. The ligation efficiency of pol μ-mediated ribonucleotide vs deoxyribonucleotide-containing repair intermediates by DNA ligase IV/XRCC4 during non-homologous end-joining. The model represents the recruitment of multi-protein complex for the repair of double-strand breaks in response to the oxidative DNA damage (8-oxodG). Based on the results observed in this study, the insertion of ribonucleotides (rATP or rCTP), not deoxyribonucleotides (dATP or dCTP), by pol μ during mutagenic bypass of 8-oxodG could lead to an efficient DNA ligation and the formation of promutagenic repair intermediates.
